# Moral foundations in autistic people and people with systemizing minds

**DOI:** 10.1186/s13229-024-00591-8

**Published:** 2024-05-14

**Authors:** Yeshaya David M. Greenberg, Rosemary Holt, Carrie Allison, Paula Smith, Robbie Newman, Theo Boardman-Pretty, Jonathan Haidt, Simon Baron-Cohen

**Affiliations:** 1 Department of Psychiatry, Autism Research Centre, University of Cambridge, Cambridge, UK; 2 CHIME Research, Center for Health Innovation, Music, and Education, Marlton, NJ USA; 3 Stern School of Business, New York University, New York, USA

**Keywords:** Autism, Moral judgements, Moral foundations theory, Empathizing-systemizing theory, Political identification, Libertarians, Empathy quotient, Systemizing quotient, Cognitive and affective empathy

## Abstract

**Background:**

Do autistic people share the same moral foundations as typical people? Here we built on two prominent theories in psychology, moral foundations theory and the empathizing–systemizing (E–S) theory, to observe the nature of morality in autistic people and systemizers.

**Methods:**

In dataset 1, we measured five foundations of moral judgements (Care, Fairness, Loyalty, Authority, and Sanctity) measured by the Moral Foundations Questionnaire (MFQ) in autistic (*n* = 307) and typical people (*n* = 415) along with their scores on the Empathy Quotient (EQ) and Systemizing Quotient (SQ). In dataset 2, we measured these same five foundations along with E–S cognitive types (previously referred to as “brain types”) in a large sample of typical people (N = 7595).

**Results:**

Autistic people scored the same on Care (i.e., concern for others) as typical people (h1). Their affective empathy (but not their cognitive empathy) scores were positively correlated with Care. Autistic people were more likely to endorse Fairness (i.e., giving people what they are owed, and treating them with justice) over Care (h2). Their systemizing scores were positively correlated with Fairness. Autistic people or those with a systemizing cognitive profile had lower scores on binding foundations: Loyalty, Authority, and Sanctity (h3). Systemizing in typical people was positively correlated with Liberty (i.e., hypervigilance against oppression), which is a sixth moral foundation (h4). Although the majority of people in all five E–S cognitive types self-identified as liberal, with a skew towards empathizing (h5), the percentage of libertarians was highest in systemizing cognitive types (h6). E–S cognitive types accounted for 2 to 3 times more variance for Care than did sex.

**Limitations:**

Our study is limited by its reliance on self-report measures and a focus on moral judgements rather than behavior or decision-making. Further, only dataset 2 measured political identification, therefore we were unable to assess politics in autistic people.

**Conclusions:**

We conclude that some moral foundations in autistic people are similar to those in typical people (despite the difficulties in social interaction that are part of autism), and some are subtly different. These subtle differences vary depending on empathizing and systemizing cognitive types.

**Supplementary Information:**

The online version contains supplementary material available at 10.1186/s13229-024-00591-8.

## Background

Do autistic people differ from typical people in their moral foundations? Autistic people experience difficulties with social communication, social interaction, and ‘theory of mind’ (also known as ‘cognitive empathy’) [1, 2] and have strengths which include attention to detail [3–5] and systemizing (analyzing and building rule-based systems) [6]. However, little is known about how these strengths and challenges contribute to their moral judgements. Research into how morality differs in autistic people has so far focused only on narrow classes of moral dilemmas, such as the Trolley dilemma [7–9] and the moral-conventional distinction [10, 11], but has not investigated a wider class of moral foundations. In this article, we address this gap by testing hypotheses from two important theories in psychology—Moral Foundations Theory (MFT) and the Empathizing-Systemizing (E–S) theory.

MFT is a theory about the moral foundations we all use to make moral judgements [12, 13]. It posits that the human mind contains five “moral foundations” upon which all individuals and cultures construct their moral codes. These are Care/harm, Fairness/cheating, Loyalty/betrayal, Authority/subversion, and Sanctity/degradation. *Care* refers to concern for the vulnerable and a dislike of harm and violence. *Fairness* involves giving people what they are owed, and treating them with justice. *Loyalty* is related to feelings of belonging to a group and prioritizing that group’s interests. *Authority* refers to ancient social primate programming to recognize a social hierarchy, including respect and deference for leaders, and a preference for order and stability. *Sanctity* involves feelings about holiness, purity, and the need to keep some things separate from others, to protect them from contamination and degradation. Individual differences on these five foundations can be measured with the Moral Foundations Questionnaire (MFQ) [12].

There are individual differences in the degree to which people and groups value each of the five moral foundations, and these give rise to different ‘moral profiles.’ Moreover, these moral profiles are closely linked to political identification. For example, those who vote for political parties on the left (e.g., Democrats in the USA; Labour in the UK) place a higher value on Care and Fairness, compared to people on the political right, and conservatives value one kind of fairness—proportionality—more than do people on the left [12]. Conversely, those who vote for right wing political parties (e.g., Republicans in the USA; Conservatives in the UK) tend to value Loyalty, Authority, and Sanctity more than do those on the left [14]. People do not all fall along a simple left–right political dimension, and by quantifying their scores on these five foundations, MFT allows us to analyze the population in a five-dimensional space. There are surely additional dimensions of morality beyond these five; for example, “Liberty/oppression” has been proposed as a 6th foundation [15]. Libertarians have been found to be more utilitarian, and they score higher than other political groups on questions about “economic liberty” and “lifestyle liberty” [15].

E–S theory posits that individuals can be classified along two dimensions of the mind [16, 17]: *Empathizing* is defined as the drive to recognize the mental states of others (‘cognitive empathy’, also known as ‘theory of mind’) and to respond to another’s mental state with an appropriate emotion (‘affective empathy’) [2]; *Systemizing* is defined as the drive to analyze or build a rule-based system [6, 18]. Both dimensions are normally distributed in the general population and have a partial biological basis in being associated with prenatal testosterone exposure and common genetic variants [19–22]. There are sex differences in empathizing and systemizing: on average, females score higher on empathizing (E), and on average, males score higher on systemizing (S) [2, 17, 18, 23]. Empathy can be measured with the Empathy Quotient (EQ) [2] and systemizing can be measured with the Systemizing Quotient (SQ) [6, 18]. E–S theory converts E and S dimensions into five distinct cognitive profiles (which we refer to as “cognitive types” rather than “brain types” as they are defined psychometrically, rather than by neural characteristics), based on the difference or D-score (S—E) between E and S. These cognitive types are Type B (balanced, where E = S), Type E (where E > S), Type S (where S > E), Extreme Type E (E >  > S) and Extreme Type S (S >  > E) (18). These cognitive type classifications have a neurobiological basis [19, 24–29]. E–S theory has been extended to understand autism [17, 30] predicting that autistic people are on average shifted towards a systemizing cognitive type (difficulties in cognitive empathy alongside average or superior systemizing). These profiles were confirmed in a study of 600,000 typical people and 36,000 autistic people [23].

It is important to emphasize that empathy is multidimensional and not unidimensional [31]. Previous research has converged to show that there are at least two latent dimensions underlie empathy, defined as cognitive empathy and affective empathy. Cognitive empathy refers to the ability to recognize or infer what another person is thinking or feeling and to predict their behavior. Cognitive empathy is also sometimes referred to as “theory of mind”, “mindreading”, or “mentalization”. Affective empathy refers to the drive to respond to another person’s mental state with an appropriate emotion. Sympathy is a specific example of empathy (with elements of both cognitive and affective empathy) and which involves understanding the suffering of another person and having an appropriate emotional response to their distress, for example of wanting to alleviate their distress [32]. Individuals and groups of individuals can have different profiles in terms of their cognitive and affective empathy. Some may have high cognitive empathy with low affective empathy, while others might have low cognitive empathy with high affective empathy. Research suggests that autistic people on average have low cognitive empathy alongside intact or elevated affective empathy. Therefore, social situations can be confusing for autistic people, yet their concern for others is often not diminished. On the other hand, the opposite profile, which is marked by high cognitive empathy with low affective, is associated with people with antisocial personality disorder or psychopathy, which explains their ability to predict and manipulate the behavior of others, but without remorse [33]. The distinction between affective and cognitive empathy is important for any investigation into morality, and we investigate empathy using these two dimensions in the present article.

MFT and E–S theories provide a basis from which hypotheses about moral judgements and autism can be made. The most relevant of the five moral foundations for E–S theory are Care and Fairness. Haidt [13] argued that the rule-based moral reasoning of Bentham and Kant was due to their extreme systemizing, in contrast to Hume’s sentimentalism, which is more rooted in empathizing. In a related vein, Baron-Cohen [33] suggested that people can develop morality by two different routes: empathizing or systemizing. To unpack this idea further, both Haidt [13] and Baron-Cohen [33], suggest that a person’s moral judgments may derive from multiple alternative cognitive processes. One such process is based on care and concern for the welfare of another person and the other is based on a rule-based understanding of the moral system. For example, in a given moral dilemma, a person who uses an empathic process might decide to break a rule if it means preventing another person from suffering. However, a person who uses a systemizing process might judge that breaking a rule could lead to injustice and argue the rule should be followed even if it entails the suffering of a specific person. According to Haidt [13] and Baron-Cohen [33], a person with more empathy is more likely to use empathy when making moral judgments, while a person with stronger systemizing is more likely to use systemizing when making moral judgment.

We can apply both empathizing and systemizing to specific moral foundations. Care, which is defined by feeling compassion and concern for others, is likely associated with an empathizing mind. Multiple studies find evidence that empathy is related to concern for others’ suffering and prosocial and helping behaviors [34, 35]. In contrast, a lack of empathy has been suggested to lead to human cruelty, violence, and criminal behavior [33]. For example, the notion that it is bad to hurt another person assumes an understanding that someone else is in pain (‘cognitive empathy’) and a desire to alleviate their distress (‘affective empathy’). High empathy would therefore be expected to lead an individual to particularly value doing no harm to others or caring for the weak and the vulnerable, as part of their moral code. This leads to the prediction that people with an empathizing profile would be more likely to endorse the moral foundation of Care. However, a deficit of empathy does not necessarily mean that an individual will act in an immoral way, because systemizing could be a separate route to making moral judgments [33].

Fairness, which is defined by rule-based reasoning is likely associated with systemizing cognitive types. People with systemizing minds may consider their emotional responses in their moral judgements, but their S > E profile is likely to override these and lead them to place a higher value on reasoning. For example, the Ten Commandments make no reference to empathy but provide a set of rules about what constitutes good behavior (‘Thou shalt not commit murder’) and could be learned as a list of dos and don’ts. Legal systems (found in all human cultures) are examples of rule-based systems to ensure constraints on behavior and which reflect the moral foundations of the lawmakers in that culture. Rule-based moral systems can be handed down (e.g., by politicians or the courts) or can be derived through systemizing (e.g., Kant urged us to apply the “categorical imperative” and act only according to maxims that we wish to be universal laws). Individuals with strong systemizing would therefore be expected to value Fairness, and again, it does not follow that those who struggle with aspects of empathy (such as autistic people) would have no basis on which to develop a moral code.

These assumptions, on how empathy is related to Care and how systemizing is related to Fairness, provide a basis for us to formulate specific hypotheses about morality in autism and E–S cognitive types. Because autistic people have intact affective empathy [36], alongside a systemizing cognitive type (Type S or Extreme Type S) [33], we hypothesized that: (h1) An autism diagnosis or a systemizing cognitive type would not differ on Care, and that: (h2) An autism diagnosis or a systemizing cognitive type predicts a higher score on Fairness.

Considering that this is the first exploration on a wider class of moral foundations in autism, we made a prediction about the other foundations (loyalty, authority, and sanctity). All five moral foundations can be categorized into two higher-order groupings: the *individualizing* foundations that comprise Care and Fairness; and the *binding* foundations that comprise Authority, Loyalty, and Sanctity [13, 37]. Haidt argues that the binding foundations were shaped in part by group-level selection pressures for a sense of “groupishness” and cohesion, which are implemented by a set of tribal sentiments [13]. Because autistic people tend to be more socially isolated (either by choice or through by exclusion from society) [38], we predicted that: (h3) An autism diagnosis or a systemizing cognitive type predicts a lower score on binding foundations: Loyalty, Authority, and Sanctity.

As was mentioned earlier, moral foundations have also been consistently found to be associated with political identification. Liberals score higher on both Care and Fairness, compared to the other three foundations, while conservatives generally score more equally across the five foundations [13]. Relevant to the study of political identification, Haidt and colleagues [15] have theorized that Liberty may exist as a sixth moral foundation. Liberty is the opposite of oppression and is characterized by hypervigilance against anyone who attempts to dominate them or others or who threatens to restrict their liberty. Endorsement of the Liberty foundation indicates a rejection of social constraints upon behavior, constraints that autistic people may feel more keenly than others. Although this sixth dimension is not captured by the original MFQ scale, it can be assessed through previously developed survey items on liberty [15]. We predicted that: (h4) An autism diagnosis or a systemizing cognitive type predicts a higher score on Liberty.

One previous study found that people who identity as libertarians value liberty more than liberals and conservatives do; Libertarians also tend to score higher than liberals and conservatives on systemizing and lower on empathizing [15]. We expected that systemizing cognitive types would be more likely to be associated with Liberty than would empathizing cognitive types. We predicted that: (h5) empathizing cognitive types would show an increased likelihood of identifying on the political left; and (h6) A systemizing cognitive type predicts an increased likelihood of identifying as a libertarian.

Throughout our investigations, we made on-average observations separately for females and males, instead of simply controlling for sex as a confounding factor. We took this approach because there is emerging evidence that the phenotype differs on average between autistic females and autistic males, in part due to societal expectations and sex differences in ‘camouflaging’ by autistic people [39]. Therefore, observing the effects separately for both females and males is more in line with the previous literature, rather than simply controlling for sex in an analysis of the entire sample with men and women combined. One exception is when we compare the variance between sex differences and E–S types in accounting for moral foundation scores—given prior evidence that E–S types are more important at predicting autistic traits than are sex differences [23], we expected a similar result when predicting moral foundations. If the results replicate across the sexes, then it would show that the differential effect of moral foundations in autistic people vs non-autistic people (or across the E–S cognitive types) is an effect of autism and (or E–S type) and not sex. Further to this point, we make observations to compare E–S types to sex explain in their explained variance for each of the five moral foundations.

## Method

### Dataset 1

#### Participants and procedures

Autistic individuals consisted of 307 participants who completed questionnaires at one of two websites (www.autismresearchcentre.com or www.cambridgepsychology.com) between 2013 and 2015. Each case indicated that they had a formal clinical diagnosis of autism. Of those who indicated a subtype, diagnoses comprised Asperger Syndrome (*n* = 103), High Functioning Autism (*n* = 16), Autism (*n* = 7), Pervasive Developmental Disorder (*n* = 3), and Other (*n* = 11). Of those who indicated, 142 (46%) were male and 166 (54%) were female, 15 (21%) were from the US and 56 (79%) were from the UK. The age ranged from 18 to 65 with a mean of 42.46 (*SD* = 12.23). Typical individuals were those who did not have a diagnosis of autism and who indicated that they did not suspect they have an autistic first degree relative. There were 415 typical participants who completed measures at www.cambridgepsychology.com. Of those who indicated, 116 (27%) were male and 298 (72%) were female, 59 (25%) were from the US and 173 (75%) were from the UK. The age ranged from 18 to 65 with a mean of 44.71 (*SD* = 11.39). There was no significant difference in age between the cases and controls (*p* = 0.073). Ethical approval was given by the Psychology Research Ethics Committee of the University of Cambridge. This study, whose study design began prior to 2013, was not pre-registered.

#### Measures

Participants completed the 32-item Moral Foundations Questionnaire (MFQ) [40]. The scale has two parts. The first 16-items measure perceptions of moral relevance (e.g., “When you decide whether something is right or wrong, to what extent do you consider whether or not someone suffered emotionally?”). Specifically, participants are asked “When you decide whether something is right or wrong, to what extent are the following considerations relevant to your thinking? Please rate each statement using this scale…” Participants are asked to rate each of the 15-items using a six-point scale that ranges from: 0 = not at all relevant (this consideration has nothing to do with my judgments of right and wrong); 1 = not very relevant; 2 = slightly relevant; 3 = somewhat relevant; 4 = very relevant; and 5 = extremely relevant (this is one of the most important factors when I judge right and wrong).

The second 16-items measure agreement with specific moral statements (e.g., “I would call some acts wrong on the grounds that they are unnatural”). Specifically, participants are asked “Please read the following sentences and indicate your agreement or disagreement.” Participants are asked to rate each item on a six-point scale ranging from: 0 = strongly disagree; 1 = moderately disagree; 2 = slightly disagree; 3 = slightly agree; 4 = moderately agree; and 5 = strongly agree. The MFQ has good psychometric properties, including strong reliability and validity, and has been shown to predict a variety of moral and political attitudes independent of political ideology [12]. Participants also provided demographic information on sex, age, and diagnosis. We did not ask participants about their political self-identification. A subsample of participants also completed the 60-item Empathy Quotient (EQ) [2], the 75-item Systemizing Quotient-Revised (SQ-R) [18], and the 60-item Autism Spectrum Quotient (AQ) [41].

The EQ is a 60-item self-report questionnaire that measures cognitive and affective components of empathy. 20 of the 60 items are filler leaving a total of 40 items that measure empathy directly. Participants are required to indicate their degree of agreement for each statement on a four-point scale (strongly disagree, slightly disagree, slightly agree, or strongly agree). For positively poled items, two points are given for strong agreement and one point is given for slight agreement. For negatively poled items, two points are given for strong disagreement and one point is given for slight disagreement.

The SQ-R is a 75-item self-report questionnaire that measure systemizing unidimensionally. Participants were required to indicate their degree of agreement for each statement on a four-point scale (strongly disagree, slightly disagree, slightly agree, or strongly agree). For positively poled items, two points are given for strong agreement and one point is given slight agreement. For negatively poled items, two points are given for strong disagreement and one point is given for slight disagreement.

The AQ is a 50-item self-report questionnaire that measures a number of autistic traits in both autistic populations and typical populations. Participants were required to indicate their degree of agreement for each statement on a four-point scale (strongly disagree, slightly disagree, slightly agree, or strongly agree). For positively poled items, one point is given for strong agreement and one point is also given slight agreement. For negatively poled items, one point is given for strong disagreement and one point is also given for slight disagreement. Though the AQ can derive latent facet scores, here we only used the total AQ score in our analysis.

### Dataset 2

#### Part 1 of dataset 2

##### Participants and procedures

A total of 7595 volunteers completed measures at www.yourmorals.org where users can take a variety of psychological measures in exchange for feedback about their scores. Of those who indicated their sex while registering at the site, 3458 (45.5%) were female and 4137 (54.5%) were male. Participants ranged from 18 to 65 with a mean of 36.63 (*SD* = 13.08). Of those who indicated, 6220 (82%) were from the United States, 318 (4%) were from Canada, and 185 (2%) were from the United Kingdom. When registering at the website, participants were asked “When it comes to politics, do you usually think of yourself as liberal, moderate, conservative, or something else?” Options available on a dropdown menu included “very liberal,” “liberal,” “slightly liberal,” “moderate/middle of the road,” “slightly conservative,” “conservative,” “very conservative,” “do not know or not political,” all in a dropdown menu that allowed us to categorize participants into liberals (75% chose one of the three liberal categories), moderates (9%), conservatives (7%), and libertarians (7%). Participants completed the MFQ [2], and a 20-item measure of the Empathy Quotient (EQ) [2] and a 20-item measure of the Systemizing Quotient-Revised (SQ-R) [18], developed in previous research [15]. The maximum score possible on the 20-item EQ is 40. Mean scores on the EQ were 23.10 (*SD* = 7.05) for females and 18.90 (*SD* = 7.22) for males. The maximum score possible on the 20-item SQ-R is 40. Mean scores on the SQ were 14.33 (*SD* = 5.15) for females and 18.03 (*SD* = 5.61) for males.

Most participants who come to YourMorals.org completed a single survey, usually the MFQ. But many participants completed several surveys, so we included all participants who had completed both the MFQ and the 40 item EQ/SQ survey. In addition, we selected 1,271 participants who had completed the EQ/SQ survey and who had also completed the 28-item Interpersonal Reactivity Index (IRI) [31]. The IRI includes two 7-item subscales of interest in the current study: empathic concern (e.g., “I often have tender, concerned feelings for people less fortunate than me”), and perspective taking (e.g., “I sometimes try to understand my friends better by imagining how things look from their perspective”). Sample characteristics for dataset 1 and dataset 2 are provided in Additional file 1: Table S1.

##### Calculating E–S cognitive types

We followed the procedure established previously for calculating E–S cognitive types based on short forms [15]. E–S cognitive type classifications are based on each individual’s D-score, which is the standardized difference of their empathizing and systemizing scores. Standardized EQ (E) and SQ-R (S) scores were calculated using calculated *T*-scores for each of the measures. The D-score is defined as: D = S − E. The cognitive types were assigned according to the percentiles on the D-axis. The lowest scoring 2.5% on the D axis are classified as Extreme Type E and the top 2.5% are classified as Extreme Type S. Those scoring between the 35th and 65th percentile is classified as Type B. Participants who scored between the 2.5th and 35th percentiles are Type E, and Type S was defined by scoring between the 65th and 97.5th percentile. The distribution of cognitive types is displayed in Additional file 1: Table S3.

#### Part 2 of dataset 2

##### Participants and procedures

A total of 805 volunteers completed measures at www.yourmorals.org. Of those who indicated their sex, 293 (38%) were female and 485 (62%) were male. Participants ranged from 18 to 65 with a mean of 32.08 (*SD* = 12.74). Of those who indicated, 575 (71%) were from the United States, 49 (5%) were from Canada, and 27 (3%) were from the United Kingdom. Participants completed the same shortened version of the EQ and SQ as in Part 1 of the dataset.

Participants completed 11 items related to the proposed sixth Liberty foundation that have been established in previous research (these items are listed in the Additional file 1) [15]. Prior research conducted a Principal Components Analysis (PCA) with varimax rotation on these 11 items yielded. Six of these items loaded greater than 0.60 on the first component, which represented concerns about economic and government liberty, which can be interpreted as a “Economic Liberty” component (e.g., ‘‘People who are successful in business have a right to enjoy their wealth as they see fit’’ and “Society works best when it lets individuals take responsibility for their own lives without telling them what to do”). Three of these items loaded greater than 0.60 on the second component, which can be interpreted as a ‘‘Lifestyle Liberty’’ component (e.g., ‘‘Everyone should be free to do as they choose, as long as they don’t infringe upon the equal freedom of others.’’). This prior research created two subscales from these items. Cronbach’s alpha was 0.81 Economic Liberty and 0.60 for Lifestyle Liberty. The correlation between the components was 0.27.

D-scores were calculated using the same method as described in Part 1 of the dataset 2. There were 119 participants in Part 2 who has also participated in Part 1 of the dataset.

### Statistical analysis

To test our six hypotheses, we conducted two independent studies and used a combination of MANOVAs, *t*-tests, linear regressions, and Pearson correlations. To be sure that differences found were not confounded with the substantial sex differences in autism diagnosis and E–S cognitive types, analyses were conducted separately for females and males. We conducted the analysis separately between the sexes (rather than moderation analyses) in consideration of prior research showing differences in the autistic phenotype across the sexes. We present the most pertinent statistics in the main text and present more detailed statistics in the Additional file 1.

## Results

In dataset 1, we examined the difference in moral foundations between autistic individuals (*n* = 307) and typical individuals (*n* = 415). Moral foundations were measured with the MFQ. Participants were recruited through the Cambridge Autism Research Database (CARD). Between-subject effects from MANOVAs showed no significant differences between autistic and typical people for Care in either sex (*p* = 0.32 for females and 0.39 for males). This is an important and unexpected finding that supports hypothesis 1. The benefit of dataset 1 is that it allowed us to observe differences in moral foundations in autistic people and typical people.

Autistic females scored higher on the Fairness foundation than typical females (*F*(1, 447) = 13.00, *p* < 0.001). This supports hypothesis 2. Autistic females scored lower on Loyalty (*F*(1, 447) = 11.93, *p* < 0.01) and Authority (*F*(1, 447) = 8.27, *p* < 0.01) than typical females, supporting hypothesis 3. There was no significant difference in any of the five moral foundations between autistic males and typical males. The difference between autistic males and typical males on Fairness was not significance (*p* = 0.08). Overall, there was no support for hypotheses 2 and 3 for males.

**Fig. 1:**
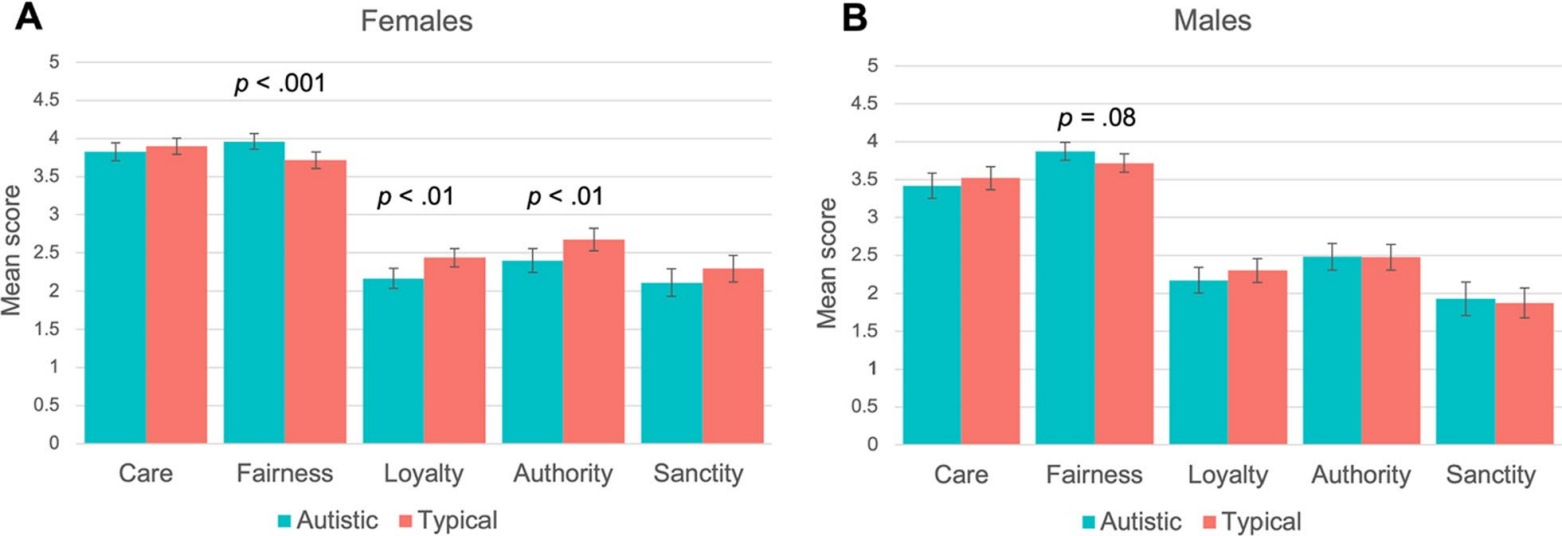
Means, separated by sex, on the five moral foundations for autistic and typical people in dataset 1. Error bars are based on 95% CIs. This figure displays mean differences between autistic people (dark green) and typical people (light green) for scores on each of the five moral foundations. Panel A displays mean differences for females and panel B displays mean differences for males. P values are presented for significant and notable differences. Mean scores on the MFQ range from 0 to 5

Figure 1 displays mean differences between autistic people and typical people across each of the five moral foundations (means, *SD*s, and Cohen’s *d* is reported in Additional file 1: Table S1). As can be seen, this set of analyses show that overall, autistic people did not substantially differ from typical people on any of the five foundations.

Given that the analysis showed little to no general differences between autistic and typical people on the five moral foundations, we decided to make more nuanced comparisons related to moral foundations. In the next step of the analysis, we took this opportunity to examine the relationship between the Care and Fairness foundations by observing a Care/Fairness “ratio” for autistic and typical participants. The Care/Fairness “ratio” is the difference in endorsement on Care compared to Fairness, and allows us to observe if one foundation is valued more than another. Paired sample *t*-tests showed that autistic females endorsed Fairness over Care (*t*(130) = 2.98, *p* < 0.01). Autistic males also endorsed Fairness over Care (*t*(134) = 6.26, *p* < 0.001). This provided further support for hypothesis 2. For comparison purposes, typical females showed the opposite profile by endorsing Care over Fairness. (*t*(160) = 1.99, *p* < 0.05). However, typical males endorsed Fairness over Care *t*(112) = 2.61, *p* < 0.05). All means, *SD*s, and Cohen’s *d*s are presented in Additional file 1: Table S2. These results showed that the differences between autistic and typical people on their endorsement of the Care and Fairness foundations are only small, if any.

We next sought to better understand the different cognitive processes (i.e., empathizing and systemizing) that people use in their moral judgements. We leveraged additional data available for a subsample of autistic and typical participants who completed the EQ and SQ. Because of the smaller sample size (*n*s = 85 autistic people and 173 typical people), instead of conducting analyses separately for females and males, we conducted linear regressions while holding sex constant. Table 1 reports Pearson correlations and Additional file 1: Table S3 reports results from linear regressions. There are several notable results. First, EQ scores were positively associated with Care for both autistic (*r* = 0.25, *p* < 0.05) and typical people (*r* = 0.28, *p* < 0.01). SQ scores were positively associated with Fairness for autistic people (*r* = 0.24, *p* < 0.05), but not typical people (*r* = − 0.04, *p* = ns). This suggests that systemizing correlates with moral foundations for autistic people, but not for typical people. Furthermore, in general, this set of analyses shows that both empathizing and systemizing are indeed involved as cognitive processes in moral foundations and that people may differ in their moral foundations based on their empathizing and systemizing traits.

**Table 1:** Pearson correlations for EQ and SQ, with the Care and Fairness foundations for autistic and typical people in dataset 1, with sex held constant

	Care		Fairness	
	Autistic	Typical	Autistic	Typical
EQ	0.25* (0.29*)	0.28** (0.21**)	− 0.03 (0.06)	0.11 (0.12)
Cognitive empathy	0.12 (0.13)	0.21** (0.14)	− 0.09 (0.04)	0.08 (0.07)
Affective empathy	0.29** (0.30**)	0.39** (0.33**)	0.09 (0.16)	0.08 (0.07)
SQ	− 0.13 (− 0.08)	− 0.30** (− 0.24*)	0.24* (0.27*)	− 0.04 (− 0.06)

*n*s = 85 for autistic and 173 for typical people

**p < 0.01, *p < 0.05. This table reports correlations for all participants (both females and males). Cell entries are Pearson corrections and entries in parentheses are partial correlations controlling for sex. Results from linear regressions where sex and all measures are held constant are reported in Additional file 1:

We previously discussed the important distinction between empathy profiles in autistic and typical people, namely, that autistic people tend to have lower cognitive empathy with intact or elevated affective empathy. This distinction might influence their moral foundations. Thus, in an exploratory analysis, we next examined the two major components of empathy (affective and cognitive empathy) and their relationship to moral foundations. Autistic people did not significantly differ from typical people on affective empathy (*p* = 0.174), however, autistic people did score significantly lower on cognitive empathy (*p* < 0.001) (Additional file 1: Table S4). This confirms that indeed in our sample, that autistic people have lower cognitive empathy with intact affective empathy, just as previous research suggests.

Given that our participants differed in terms of their cognitive and affective empathy, we next analyzed how these two different components of empathy are associated with moral foundations. As seen in Table 1, affective empathy was positively associated with Care for autistic (*r* = 0.29, *p* < 0.01) and typical people (*r* = 0.39, *p* < 0.01). Cognitive empathy was not significantly correlated with Care for autistic people (*r* = 0.12, *p* = ns), but was significantly correlated for typical people (*r* = 0.28, *p* < 0.01). This suggests that for autistic people, moral foundations are influenced more by their affective empathy levels than cognitive empathy levels. For typical people, their moral foundations seem to be influenced by both empathy components.

We next investigated if the number of autistic traits a person has is associated with their moral foundations. Results from Pearson correlations for the Autism Spectrum Quotient (AQ) and the social skills component of the EQ are reported in Additional file 1: Tables S3 and S5). The results were found to be largely non-significant which further provides evidence for our finding that autistic people and typical people do not differ much in terms of their moral foundations (i.e., if there were substantial differences, we would expect large associations with AQ scores).

To summarize, there were five important findings from dataset 1. First, autistic people overall had similar moral foundation scores to typical people. Second, autistic people scored the same on Care and this was true for both females and males. Third, the significant differences that were found were relatively small and showed that autistic females score higher on Fairness than typical females. In terms of the Care/Fairness ratio, both autistic females and males endorsed Fairness over Care. This suggests that Fairness may play a larger role in the moral judgements in autistic people than in typical people. Fourth, empathy is correlated with Care in both autistic and typical people. Fifth, systemizing correlated with Fairness in autistic people but not in typical people.

These correlations between moral foundations and both empathizing and systemizing in dataset 1 confirm our hypothesis that empathy and systemizing are two different constructs that influence the way in which people cognitively process moral judgments. However, the subsample size of people who completed the EQ and SQ in dataset 1 was relatively small. The sample size did not allow us to make observations about E–S types, which considers the standardized difference between scores on the EQ and SQ (Introduction and Methods). Therefore, we sought to leverage a larger dataset that included EQ and SQ scores with moral foundation scores. Furthermore, dataset 1 had no data on politics—how a person identifies politically is an extension of their moral judgments, and as discussed in the Introduction, indeed political identification and moral foundation scores.

To address the gap from dataset 1, dataset 2 was collected from www.YourMorals.org and consisted of more than 7000 typical participants who completed the MFQ and shortened versions of the EQ and SQ (Methods). This enabled us to observe how the five E–S cognitive types in the general population are associated with the five moral foundations. We calculated D-scores for each participant which is the basis of E–S classifications. D-scores are the standardized difference between a person’s scores on the EQ and SQ (see Methods). High D-scores indicate systemizing and low D-scores indicate empathizing. E–S cognitive type classifications are based on D-scores. The distribution of E–S types in dataset 2 is presented in Additional file 1: Table S6. The dataset did not ask participants about their clinical diagnoses so we were unable to identify participants who may have an autism diagnosis.

In the first stage of the analysis in dataset 2, we aimed to see if there was an association between D-scores and moral foundation scores. Thus, we calculated Pearson correlations between D-scores and scores on each of the five moral foundations (Table 2). As can be seen, D-scores were negatively correlated with Care for both females (*r* = − 0.26, *p* < 0.001) and males (*r* = − 0.24, *p* < 0.001). However, D-scores were also negatively correlated with Fairness for both females (*r* = − 0.08, *p* < 0.001) and males (*r* = − 0.10, *p* < 0.001), albeit to a lesser degree than Care. This was contrary to hypothesis 2, which predicted D-scores would be positively corelated with Fairness.

**Table 2:** Pearson correlations between D-scores, EQ and SQ scores, and moral foundations in the non-clinical sample in dataset 2

	Females (*N* = 3458)			Males (*N* = 4137)		
	D-score	EQ	SQ	D-score	EQ	SQ
Care	− 0.24**	0.29**	− 0.09**	− 0.24**	0.28**	− 0.04**
Fairness	− 0.10**	0.13**	− 0.02	− 0.08**	0.13**	0.02
Loyalty	0.00	0.02	0.02	− 0.01	0.01	0.01
Authority	0.00	0.02	0.01	0.00	0.00	0.01
Sanctity	− 0.06**	0.03	− 0.06**	− 0.04	0.02	− 0.03

Pearson correlations between D-scores, EQ and SQ scores, and each of the five moral foundations in the non-clinical sample. *p < 0.01, **p < 0.001

Since low D-scores indicate a drive to empathize while high D-scores indicate a drive to systemizing, the correlational results thus far from dataset 2 seem to suggest that Fairness scores are accounted for more by empathy than systemizing (logic and reasoning). To further examine if this is the case, we correlated each of the five moral foundations with EQ and SQ scores. The results showed the same pattern. Care was positively correlated with EQ and negatively correlated with SQ, for both females and males. Fairness was positively correlated with EQ for both sexes, but had an almost zero correlation with SQ for both females and males (see Table 2). This further contrasts hypothesis 2 and suggests that responses to Fairness items on the MFQ may not be driven by systemizing or reasoning more generally, in the general population, and is consistent with the results from the typical sample in dataset 1. Therefore, it appears that systemizing is only correlated with Fairness scores in autistic people, but not in typical people. Therefore, autistic people may rely more on their systemizing when making moral judgments about Fairness (as seen in dataset 1), but typical people do not to the same extent (as seen in datasets 1 and 2). This provides more evidence that even though autistic people and typical people may end up making similar moral judgments, the cognitive processes used to make those judgments may be different.

**Fig. 2:**
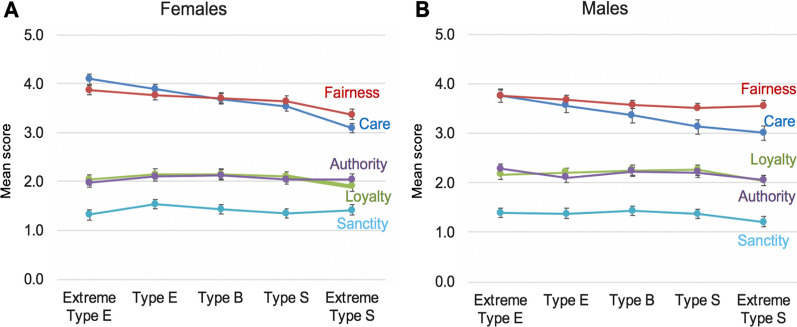
Means, separated by cognitive type and sex, on the five moral foundations in dataset 2. Error bars are based on 95% CIs. Panel **A** displays results for females and panel **B** displays results for males

We next made more nuanced observations of moral foundations and empathizing and systemizing. Rather than relying on just D-scores alone, we observed how five different classifications of empathizing and systemizing are associated with moral foundations. Toward that end, in the second stage of analysis in dataset 2, we divided the sample into the five E–S types based on their D-scores. We then conducted MANOVAs separately for females and males to examine differences in moral foundation scores between the E–S types (see Additional file 1: Tables S7–S9 for the results, including all *M*s, *SD*s, effect sizes, and *p* values, and results from post-hoc tests). Results from the MANOVAs showed that empathizing types (Extreme Type E and Type E) scored higher on Care than systemizing types (Type S and Extreme Type S), for both females and males. This provided support for hypothesis 1. However, empathizing types also scored higher on Fairness than systemizing types for both females and males. This contradicts hypothesis 2. Figure 2 displays mean scores on each of the five moral foundations by each of the five cognitive types.

In dataset 1, we made observations about the nuanced relationships between Care and Fairness by observing the Care/Fairness ratio. Thus, in the third stage of the analysis of dataset 2, we examined the Care/Fairness ratio for each E–S type. Paired-sample *t*-tests showed that females with empathizing types (Type E and Extreme Type E) endorsed Care over Fairness (*p*s < 0.001). Males who were Extreme Type E endorsed Care and Fairness equally (*p* = 1.00), but males with a Type E endorsed Fairness over Care (*p* < 0.001). Both females and males who were Type S (extreme Type S) endorsed Fairness over Care (*p* < 0.001). Females who were Type S scored descriptively higher on Fairness than Care to a degree that approached, but was not significant (*p* = 0.08). Males who were Extreme Type S endorsed Fairness significantly more than Care (*p* < 0.001) (*M*s and *SD*s are reported in Additional file 1: Table S9).

We were surprised that systemizing (as measured by the SQ) and systemizing types, were not more strongly associated to Fairness in the general population, as our second hypothesis had predicted. Rather, it appeared that lower empathizing may be contributing to a person’s Care and Fairness scores. Accordingly, to gain a better understanding of the role of empathizing and moral foundation scores, we conducted exploratory linear regressions, using SQ scores, cognitive empathy (measured via the perspective taking facet of the IRI) and affective empathy (measured via the empathic concern facet of the IRI) as predictors of Care (*r*^2^ = 0.27 for females and 0.31 for males) and Fairness (*r*^2^ = 0.09 for females and 0.19 for males) (Additional file 1: Table S10). For Care, affective empathy was the most significant predictor for females (*β* = 0.52, *p* < 0.001) and males (*β* = 0.56, *p* < 0.001). For males, SQ scores were a negative predictor of Care (*β* = − 0.14, *p* < 0.001). For Fairness, affective empathy was the only significant positive predictor for both females (*β* = 0.31, *p* < 0.001) and males (*β* = 0.45, *p* < 0.001). (For supplementary analysis, see Additional File 1: Fig S1). These analyses further showed that the affective component of empathy is contributing most to Care and Fairness in typical people, which is consistent with findings from dataset 1, and also that systemizing is not correlated to Fairness scores for typical people, which is also consistent with dataset 1. In other words, affective empathy levels appear to be more influential in their moral foundations of Care and Fairness than cognitive empathy, and systemizing levels.

In the fourth stage of the analysis in dataset 2, we extended the results by leveraging data about an additional 6th moral foundation proposed by Haidt (2012): Liberty. We leveraged new data from the YourMorals.org database and included participants who completed a 37-item survey that included 11 items that had been developed to explore Liberty, divided into two facets: Economic Liberty (e.g., “People who are successful in business have a right to enjoy their wealth as they see fit”) and Lifestyle Liberty (e.g., “People should be free to decide what group norms or traditions they want to follow”. See Methods and Additional File 1). We included only the 805 participants who had completed that survey and who had also completed the EQ and SQ questionnaires (see Methods). We correlated D-scores, EQ scores and SQ scores with the two liberty facets. We focused on D-scores rather than distinctions between the five cognitive types since the second part of the dataset had a smaller sample size (which would yield very small *n*s for the extreme cognitive types), compared to the first part of the dataset. As can be seen in Table 3, D-scores (high scores indicate systemizing and low scores indicate empathizing cognitive types) were positively correlated with both subsets of Liberty items. EQ scores were negatively correlated with Lifestyle Liberty and Economic Liberty. Surprisingly, SQ was not correlated with either of those two facets. The pattern of correlations was largely consistent for both female and male participants.

**Table 3:** Pearson correlations for D-scores, EQ, and SQ, with four facets of the Liberty foundation

Liberty facet	D		EQ		SQ	
	Females	Males	Females	Males	Females	Males
Lifestyle liberty	0.16**	0.07	− 0.14*	− 0.08	0.10	0.03
Economic liberty	0.22**	0.18**	− 0.28**	− 0.22**	0.08	0.04

ns = 293 for females and 485 males

*p < 0.05, **p < 0.01

**Fig. 3:**
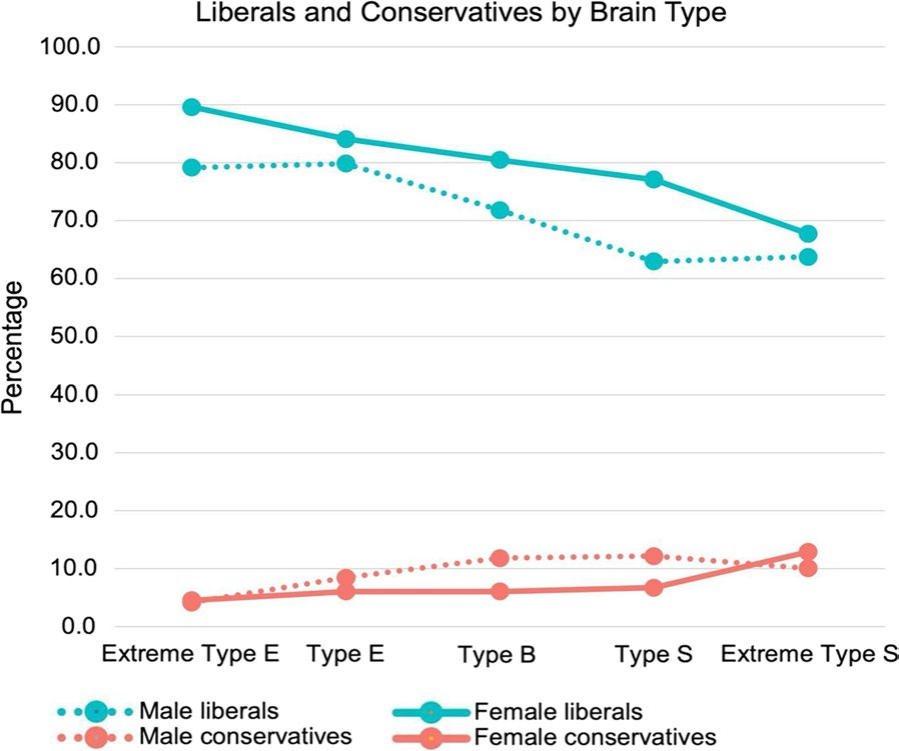
The percentage of self-identified liberals and conservatives for each cognitive type in Study 2. Blue solid lines indicate female liberals, blue dotted lines indicate male liberals, red solid lines indicate female conservatives, and red dotted lines indicate female conservatives

In the next stage of analysis, we addressed the following question: Do associations found for empathizing and systemizing and moral foundations also manifest in political identification? To address this question, we analyzed political self-identification in the dataset 2. D-scores were positively correlated with a 7-point scale for the liberalism-conservatism spectrum for females (*r* = 0.07, *p* < 0.001) and males (*r* = 0.09, *p* < 0.0001), meaning that conservatives had slightly higher D scores. This relationship is more clearly illustrated in Fig. 3, where we have plotted the percentage of men and women who self-identified as liberal or as conservative, as a function of E–S type. While most of the sample in dataset 2 identifies as liberal, the percentage of liberals decreases as we move from the drive to empathize to the drive to systemize. The opposite trend occurs for conservatives.

**Fig. 4:**
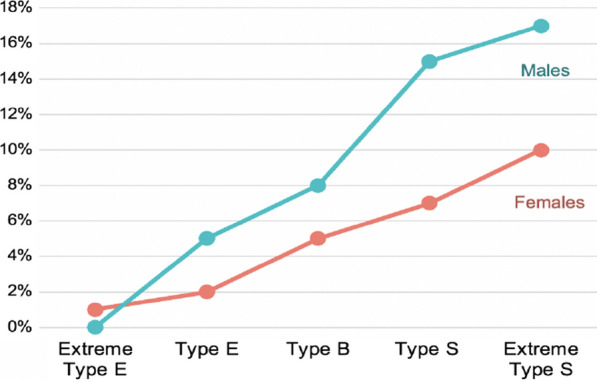
Percentage of self-identified libertarians for E–S cognitive type in dataset 2. The red line indicates females and the blue line indicates males

A unique aspect of dataset 2 is that it does not force participants to place themselves on the left–right axis (from liberal to conservative), unlike most surveys. Rather, it provides the option to identify as “libertarian.” We measured how D-scores differ across political self-identification for four categories: liberal, moderative, conservative, and libertarian. ANOVAs showed that libertarians had significantly higher D-scores than the three other political categories for females (*F*(3, 3323) = 22.17, *p* < 0.0001) and males (*F*(3, 3862) = 36.24, *p* 0.0001) (*M*s, *SD*s and results from post hoc tests are in Additional file 1: Tables S11-S12). This relationship is most clearly illustrated in Fig. 4, which plots the percentage of participants with each cognitive type who self-identified as libertarian. Libertarians are almost non-existent among those who are extreme Type E, but are common among those who are extreme Type S: 10% of women and 17% of men. More specifically, the percentage of libertarians differed significantly across the five cognitive types, for both females (*χ*^2^ (4, 3458) = 46.39, *p* = 2.04 × 10^–9^) and males (*χ*^2^ (4, 4137) = 75.98, *p* = 1.24 × 10^–15^). The majority of people in each E–S type identified as liberal (68% for Extreme Type S to 90% for Extreme Type E in females, and 64% in Extreme Type S to 80% for Type E in males). However, libertarians within each cognitive type increased across the cognitive types reaching its highest proportion in Extreme Type S (10% for females and 17% for males) (Fig. 4) (percentages for political identification and E–S types are in Additional file 1: Table S13).

In the last stage of the analysis, we made observations about the contributions of sex differences compared to E–S cognitive types in predicting moral foundations across both datasets. Toward that end, we conducted stepwise linear regressions in both datasets. In dataset 1, we entered group type (autism and typical) in the first step as a control variable. Then we entered sex (female and male) in the second step, and D-score (the basis of E–S types) in the third step, and observed the amount of variance that D-scores explained over and above sex in predicting scores on the Care foundation. We then conducted a separate regression, this time with D-scores entered in the second step and sex entered in the third step. We repeated this set of analyses separately for all five moral foundations in dataset 1. Results showed that D-score accounted for a 7% increase in the variance explained (*r*^2^ = 0.10, change in *r*^2^ = 0.07, change in *F* change = 17.94, *p* < 0.001), which was an improvement from *r*^2^ = 0.03 using sex without D-scores. This suggests that D-scores account for at least three times more variance in the Care foundation than does sex. Sex did not significantly improve the model above and beyond D-scores, suggesting that the differences we observe between the sexes on the Care foundation may really be about differences in E–S types. There were no significant differences observed between D-scores and sex in predicting Fairness, Authority, or Loyalty. Sex did predict Sanctity above and beyond D-scores *(r*^*2*^ = 0.03, change in *r*^2^ = 0.02, change in *F* = 5.74, *p* < 0.05), but D-scores did not improve the model above sex (change in *r*^2^ = 0.001, change in *F* = 0.23, *p* = 0.63).

We conducted the same analyses in dataset 2, however, without included group type as a controlling variable since there was no diagnostic data in dataset 2. The results largely replicated. D-scores predicted Care above and beyond sex (*r*^2^ = 0.14, change in *r*^2^ = 0.05, change in *F* = 432.82, *p* < 0.001). In this dataset, sex also contributed variance above and beyond D-scores (*r*^2^ = 0.14, change in *r*^2^ = 0.03, change in *F* = 292.59, *p* < 0.001). As can be seen in comparing the changes in *r*2, D-scores accounted for almost two times more variance than did sex. Though D-scores and sex contributed significantly to the models for Fairness, Authority, Loyalty, and Sanctity foundations, they were not differences greater than 1% in the increased proportions of variance between sex and D-scores, suggesting that they had relatively similar contributions to those foundations. Overall, these sets of regression analyses from both datasets show that D-scores and sex contribute relatively equally to moral foundations, except for the case of Care, where D-scores account for two to three times more variance than does sex.

## Discussion

We provide the first broad portrait of moral foundations in autistic people and people with systemizing minds. To do this, we tested six hypotheses based on two important theories in psychology—MFT and E–S theory—and leveraged two unique datasets––a dataset of autistic and typical people, and a large non-clinical dataset. We found that autistic people or those with systemizing minds do not differ much from others in terms of their moral judgments, as measured by the Moral Foundations Questionnaire. There are, however, several subtle differences, and a substantial difference in political preferences.

In dataset 1, scores on the Moral Foundations Questionnaire did not differ substantially between people with and without an autism diagnosis. Those differences that did emerge showed that autistic people tend to place a slightly higher endorsement on Fairness, compared to typical people. Importantly, autistic people did not differ in Care scores compared to typical people. This shows, as we have previously argued, that autistic people are very different than psychopaths or those with antisocial personality disorder, who would be expected to score lower on Care [33, 42]. Other populations, including those with borderline personality disorder (only when they feel threatened), and those with narcissistic personality disorder, have also been found to have lower degrees of empathy [33]. Autistic people’s responses on items about Care were not lower, despite having difficulties with social communication and with aspects of social cognition, including theory of mind (or cognitive empathy). Empathizing scores were correlated with the Care foundation for autistic and typical people, while systemizing scores were correlated with the Fairness foundation for autistic people (but not for typical people). Exploratory analyses showed that Care scores among autistic people were associated with affective empathy but not with cognitive empathy.

In dataset 2, among a large non-clinical sample, empathizing scores were correlated with the Care foundation, as predicted. Once again, fairness scores were not correlated with systemizing scores. People with empathizing cognitive types scored higher on Care than did people with systemizing cognitive types, and people with systemizing cognitive types scored higher on Fairness than did people with empathizing cognitive types. As in dataset 1, the affective component of empathy was more closely associated with Care than was the cognitive component of empathy.

In contrast to the small differences in moral foundations, we found larger differences in political attitudes related to cognitive types. First, we found that systemizing scores were correlated with concerns about Liberty, a proposed sixth foundation. Second, we found that people with systemizing cognitive types were the most likely to identify as libertarians. This finding is consistent with previous research examining the psychology of libertarians, who score higher than both liberals and conservatives on measures of traits related to reasoning, such as preference for utilitarian solutions (on moral dilemmas), while scoring lower than liberals and conservatives on traits related to group-identity, such as collectivism and identification with all of humanity [40]. For those that placed themselves along the traditional liberal-conservative dimension, people with more systemizing cognitive types were slightly more conservative.

Autistic individuals did not score lower on Care than typical individuals in dataset 1. This is consistent with the idea that although autistic people have deficits in cognitive empathy, their affective empathy—responding with care and concern for others—is intact. In support of this, in both Studies 1 and 2, affective empathy was correlated more highly with Care than was cognitive empathy. This suggests that the use of cognitive empathy in real time is independent of affective empathy and the moral foundation of Care.

Moral judgements are complex, and this complexity is seen in our findings for Fairness. Specifically, autistic people scored higher on Fairness than did typical people in dataset 1 (significant for females, and this approached significance for males) and people with systemizing cognitive types scored higher on Fairness than did people with empathizing cognitive types in dataset 2. This raises a question: are heightened levels in Fairness due to (1) lower empathizing among autistic people and people with systemizing cognitive types, (2) strengths in systemizing in autistic people and people with systemizing cognitive types, or (3) a combination of these? Given that the Care foundation was intact in the autistic sample in dataset 1, this would lead us to assume that the heightened scores on Fairness, in autism at least, is due to systemizing. However, if this were true we would have expected systemizing to be correlated with Fairness, but this was not the case in dataset 2. In fact, empathizing was also found to be much more strongly correlated with Fairness than was systemizing. Therefore, for dataset 2, it appears that the heightened scores on Fairness among systemizers (compared to empathizers) is due to the lower levels of empathizing in systemizing cognitive types. Together, this suggests that perhaps the heightened Fairness in autism is due to their systemizing, but the heightened Fairness in people with systemizing cognitive types in the general population is due to low empathizing.

In dataset 2, the differences across cognitive types were generally small, with the exception of a larger difference on the Care foundation, for both sexes. Given the well documented differences in social cognition between autistic individuals and typical individuals, we might have expected larger differences, particularly for the three foundations whose evolutionary function is argued to motivate people to attend to group cohesion and intergroup competition (Loyalty, Authority, and Sanctity). The failure to find large differences in moral judgment between autistic and typical people replicates and extends recent findings on moral dilemma tasks [43]. It also resembles an older debate in moral psychology, over whether men on average have a stronger “ethic of justice” [44] while women on average have a stronger “ethic of care” [45]. In fact, sex differences in moral *judgment* are few and far between [46] even though sex differences in moral *behavior*, such as devoting time to taking care of elderly parents or committing violent acts, are often found.

We suggest that the “ethic of justice” and “ethic of care” are not necessarily linked to sex directly, but rather to E–S cognitive types. We tested this statistically and found that E–S types and sex accounted for similar proportions of variance in explaining four of the moral foundations (Fairness, Authority, Loyalty, and Sanctity). For the Care foundation, however, we found that E–S types accounted for three times more variance than did sex in dataset 1, and almost two times more variance than sex in dataset 2. In fact, in dataset 1, sex did not account for any significant variance above and beyond E–S types when it came to the Care Foundation. This suggests that indeed, the differences proposed and observed by Gilligan for the ‘ethic of care’ might have more to do with differences in empathizing and systemizing than with sex. A limitation of our conclusion about E–S types and sex is the reliance on self-report measures (which we will discuss further in the Limitations below). This leads to a prediction: if a study used Kohlberg’s [43] moral judgment dilemmas and measures of Gilligan’s ethic of care, along with a measure of EQ and SQ, it would find that within each sex, people with an Extreme Type E cognitive type would score higher on measures of the ethic of care compared to people with an Extreme Type S cognitive type. In contrast, the opposite pattern would be found on Kohlberg’s measures of justice reasoning. We predict that cognitive type would be a more important individual difference in moral judgment than is biological sex or gender identification when using moral dilemmas, just as they were found for the Care foundation in our investigation of moral foundations.

## Limitations

The present research has several limitations. First, our studies focused exclusively on moral judgments. Future research should investigate moral behavior and decision-making. Indeed, some prior neurobiological work found no behavioral differences between autistic people and typical people in their moral decision making, yet found significant neurobiological differences between autistic people and controls while responding to written moral dilemmas [47]. Furthermore, it may be that autistic people do not diverge significantly from others when judging situations that do not elicit strong emotion, but the divergence increases as situations include more extreme or emotional content. For example, a recent study showed that autistic adolescents and adults demonstrate different moral decision-making from typical controls for extreme life-or-death dilemmas [48]. Neuroimaging and experimental studies are needed to understand the behavioral manifestations of the neurobiological differences that underpin moral decisions.

A second limitation of our work is that the dataset in dataset 1 did not assess political identification in autistic people. Although we established the moral profile in self-identified autistic people (in dataset 1) and we assessed the political identities of people across the five cognitive types (in dataset 2), there is a need for a study that asks a large sample of self-identified autistic people to report their political identities. Third, data in both studies were based on volunteer samples who could access the internet, which limits generalizability of the results to autistic people who have co-occurring learning difficulties or intellectual disability. A fourth limitation is that previous theory and research suggests that individuals who score high on psychopathy have difficulties with affective empathy but have intact or elevated cognitive empathy—the opposite profile to autism [49, 50]. However, our study did not ask if the person had a diagnosis of a personality disorder. In terms of morality, psychopaths score low on both Care and Fairness and are willing to violate moral concerns of any kind [42]. This contrasts with our findings with autistic people and people with systemizing brains. This is an important clinical distinction that should be examined in future research.

## Conclusions

In conclusion, the present studies are the first to assess the broad domain of morality among autistic people and people with a systemizing cognitive type. While we found a variety of differences that reached statistical significance, most of the differences were small in magnitude, leading to the conclusion that moral foundations are not radically different in autistic people. The preference by autistic people for Fairness over Care, and their attraction to libertarian politics, are both consistent with conceptualizations of autism involving a preference for systemizing over empathizing. In this way our study contributes to the growing body of research documenting links from personality traits to moral and political world views [12].

### Supplementary Information

**Additional file 1**. (1) The assessment items for the sixth foundation called, liberty; (2) Supplemental figures S1 to S2, and (3) Supplemental tables S1 to S12.

## Data Availability

Volunteers in the Cambridge Autism Research Database (CARD) (dataset 1) did not consent for their data to be deposited in an Open Access archive. However, the CARD Management Committee considers requests by researchers for specific parts of the database (in anonymized form) to test specific hypotheses (please contact research@autismresearchcentre.com. The anonymized dataset of the dataset 2 can be obtained from Jonathan Haidt by request from researchers with a university appointment.

## Abbreviations

AQ: Autism spectrum quotient
EQ: Empathy quotient
Extreme Type E: Extreme empathizing cognitive type
Extreme Type S: Extreme systemizing cognitive type
MFQ: Moral Foundations Questionnaire
SQ: Systemizing quotient
SQ-R: Systemizing quotient-revised
Type B: Balanced cognitive type
Type E: Empathizing cognitive type
Type S: Systemizing cognitive type
