# Supplementary material for: Moral foundations in autistic people and people with systemizing minds
Source: Mol Autism. 2024 May 14;15:20. doi: 10.1186/s13229-024-00591-8 (PMC11092219; doi:10.1186/s13229-024-00591-8)
Supplement: Supplementary file 1 — Additional file 1. (1) The assessment items for the sixth foundation called, liberty; (2) Supplemental figures S1 to S2, and (3) Supplemental tables S1 to S12. [file 13229_2024_591_MOESM1_ESM.docx]

**Supplemental Material**

Contents:

Liberty Foundation Items

Supplementary Figs S1 – S2

Supplementary Table S1 – S13

Liberty Foundation Items

Economic Liberty Items:

- Whether or not private property was respected (relevance rating)
- People who are successful in business have a right to enjoy their wealth as they see fit
- Society works best when it lets individuals take responsibility for their own lives without telling them what to do.
- The government interferes far too much in our everyday lives.
- The government should do more to advance the common good, even if that means limiting the freedom and choices of individuals. (Reverse scored)
- Property owners should be allowed to develop their land or build their homes in any way they choose, as long as they don't endanger their neighbors.
- I want my nation to stay clear of treaties that will limit our freedom to act in our own interest.
- I want my nation to join international treaties that will benefit the world, even when those treaties will require my nation to give up some sovereignty and control. (Reverse scored)

Lifestyle Liberty Items:

- Whether or not everyone was free to do as they wanted. (relevance rating)
- I think everyone should be free to do as they choose, so long as they don't infringe upon the equal freedom of others.
- People should be free to decide what group norms or traditions they themselves want to follow.

**Supplementary Figs S1 – S2**

**Fig. S1**

**Supplementary Tables S1 – S13**

**Table S1. Sample characteristics for dataset 1 and dataset 2.**

|  | Dataset 1 | | Dataset 2 | |
| --- | --- | --- | --- | --- |
|  | Autistic group | Typical group | Part 1 | Part 2 |
| *n* | 307 | 415 | 7,595 | 805 |
| Female (%) | 54% | 27% | 45.5%) | 38% |
| Male (%) | 46% | 72% | 54.5%) | 62% |
| Age (M, SD) | 42.46 (*SD* = 12.23) | 44.71 (*SD* = 11.39) | 36.63 (*SD* = 13.08). | 32.08 (*SD* = 12.74). |
| Ethnicity (%white) | 85% | 86% | - | - |
| Education (% did not complete high school (or A-levels) | 7.9 | 3.7 | 2.2 | 8.7 |
| Education (% completed high school (or A-levels)) | 20.8 | 23.4 | 26.1 | 29.6 |
| Education (% completed undergraduate degree) | 30.7 | 37.2 | 36.7 | 37.8 |
| Education (% completed postgraduate degree) | 40.6 | 35.7 | 34.9 | 23.9 |
| %UK | 79% | 75% | 2% | 3% |
| %US | 21% | 25% | 82% | 71% |
| English as native language | - | - | 90% | - |

**Table S2. Means, SDs, and effect sizes for across five moral foundations in cases and controls in dataset 2.**

|  | | Care | | | | Fairness | | |  | Loyalty | | | | Authority | | | | Sanctity | | | |
| --- | --- | --- | --- | --- | --- | --- | --- | --- | --- | --- | --- | --- | --- | --- | --- | --- | --- | --- | --- | --- | --- |
|  | *N* | Mean | *SD* | *p* | Cohen's *d* | Mean | *SD* | *p* | Cohen's *d* | Mean | *SD* | *p* | Cohen's *d* | Mean | *SD* | *p* | Cohen's *d* | Mean | *SD* | *p* | Cohen's *d* |
| Male cases | 135 | 3.42 | .99 | .381 | .11 | 3.873 | .72 | .079 | .23 | 2.17 | .98 | .287 | .14 | 2.48 | 1.04 | .968 | .01 | 1.93 | 1.32 | .704 | .05 |
| Male controls | 113 | 3.52 | .82 |  |  | 3.717 | .66 |  |  | 2.30 | .84 |  |  | 2.48 | .92 |  |  | 1.87 | 1.07 |  |  |
| Female cases | 161 | 3.83 | .76 | .321 | .10 | 3.962 | .67 | .000 | .36 | 2.17 | .83 | .001 | .34 | 2.40 | 1.03 | .004 | .28 | 2.11 | 1.15 | .097 | .16 |
| Female controls | 287 | 3.90 | .69 |  |  | 3.715 | .71 |  |  | 2.44 | .78 |  |  | 2.67 | .94 |  |  | 2.29 | 1.10 |  |  |

*This table provides the means, SDs, the effect size estimates (Cohen's d), and the associated p value (t-test) for the five moral foundations separately for cases and controls in Study 1.*

**Table S3. Results from linear regressions in Study 1 were EQ, SQ, and AQ predicted Care, and Fairness for autistic and non-autistic people in dataset 1.**

| Autistic (n = 85) | | | | | | | | | | | | | | | | |
| --- | --- | --- | --- | --- | --- | --- | --- | --- | --- | --- | --- | --- | --- | --- | --- | --- |
| Variables | Care | | | | | | | | Fairness | | | | | | | |
|  | r | Unstandardized B | STE | Standardized Beta | t | p | 95% CI (low) | 95% CI (high) | r | Unstandardized B | STE | Standardized Beta | t | p | 95% CI (low) | 95% CI (high) |
| (Constant) | - | 1.66 | .98 |  | 1.69 | .096 | -.30 | 3.61 | - | 2.51 | .79 |  | 3.17 | .002 | .93 | 4.09 |
| Sex (1 = male; 2 = female) | - | .10 | .18 | .06 | .58 | .565 | -.25 | .45 | - | .11 | .14 | .09 | .77 | .444 | -.17 | .39 |
| EQ | .25* | .03 | .01 | .51 | 2.59 | .012 | .01 | .06 | -.03 | .01 | .01 | .25 | 1.22 | .225 | -.01 | .03 |
| SQ | -.13 | .00 | .01 | -.03 | -.18 | .856 | -.01 | .01 | .24* | .01 | .00 | .34 | 2.35 | .022 | .00 | .02 |
| AQ | -.11 | .03 | .02 | .35 | 1.70 | .093 | -.01 | .07 | .10 | .01 | .02 | .11 | .51 | .611 | -.02 | .04 |
|  | *r* = .32; *r*2.= .10 | | | | | | | | *r* = .29; *r*2.= .09 | | | | | | | |
| Non-autistic (n = 173) | | | | | | | | | | | | | | | | |
|  | r | Unstandardized B | STE | Standardized Beta | t | p | 95% CI (low) | 95% CI (high) | r | Unstandardized B | STE | Standardized Beta | t | p | 95% CI (low) | 95% CI (high) |
| (Constant) | - | 2.84 | .46 |  | 6.16 | .000 | 1.93 | 3.75 | - | 3.38 | .47 |  | 7.27 | .000 | 2.46 | 4.30 |
| Sex (1 = male; 2 = female) | - | .17 | .13 | .10 | 1.34 | .182 | -.08 | .43 | - | -.05 | .13 | -.03 | -.39 | .695 | -.31 | .21 |
| EQ | .28** | .02 | .01 | .32 | 3.05 | .003 | .01 | .03 | .11 | .01 | .01 | .14 | 1.27 | .206 | .00 | .02 |
| SQ | -.30** | -.01 | .00 | -.24 | -3.08 | .002 | -.02 | .00 | -.04 | .00 | .00 | -.03 | -.35 | .729 | -.01 | .01 |
| AQ | -.15 | .02 | .01 | .20 | 1.84 | .067 | .00 | .04 | -.06 | .00 | .01 | .04 | .38 | .706 | -.02 | .02 |
|  | *r* = .40; *r*2.= .16 | | | | | | | | *r* = .12; *r*2.= .01 | | | | | | | |

*This table shows results from linear regressions. Care scores were the DV and sex, EQ, SQ, and AQ were the IVs (model 1) and where Fairness scores were the DV and sex, EQ, SQ, and AQ were the IVs (model 2). Separate regressions were performed for autistic people and non-autistic people.*

**Table S4. Mean differences on three components of the EQ between autistic people and non-autistic people in dataset 1.**

|  | | | | | Levene's Test for Equality of Variances | | t-test for Equality of Means | | | | | | |
| --- | --- | --- | --- | --- | --- | --- | --- | --- | --- | --- | --- | --- | --- |
|  | Group | Mean | *SD* | Cohen's *d* | *F* | *p* | *t* | df | Sig. (2-tailed) | Mean Difference | Std. Error Difference | 95% Confidence Interval of the Difference | |
| Cognitive empathy | autistic | 1.21 | 2.25 | 1.86 | 21.60 | 0.00 | -13.49 | 256.00 | 0.00 | -4.83 | 0.36 | -5.53 | -4.12 |
|  | non-autistic | 6.04 | 2.90 |  |  |  |  |  |  |  |  |  |  |
| Affective empathy | autistic | 2.67 | 2.32 | 1.27 | 1.85 | 0.17 | -9.44 | 256.00 | 0.00 | -3.09 | 0.33 | -3.73 | -2.44 |
|  | non-autistic | 5.76 | 2.54 |  |  |  |  |  |  |  |  |  |  |
| Social skills | autistic | 1.27 | 1.83 | 1.93 | 28.98 | 0.00 | -13.72 | 256.00 | 0.00 | -4.53 | 0.33 | -5.18 | -3.88 |
|  | non-autistic | 5.80 | 2.76 |  |  |  |  |  |  |  |  |  |  |

**Table S5. Pearson product moment correlations for the social skills component of the EQ in dataset 1.**

|  | Care | | Fairness | |
| --- | --- | --- | --- | --- |
|  | Autistic | Non-autistic | Autistic | Non-autistic |
| Social Skills component of EQ | .08 | .07 | -.05 | .07 |

*All results in the table are not significant.*

**Table S6. Frequency distribution of cognitive types in dataset 2.**

|  | Males | | Females | |
| --- | --- | --- | --- | --- |
|  | *N* | % | *N* | % |
| Extreme Type E | 24 | 0.60 | 159 | 4.60 |
| Type E | 772 | 18.70 | 1,695 | 49.00 |
| Type B | 1,295 | 31.30 | 983 | 28.40 |
| Type S | 1,882 | 45.50 | 588 | 17.00 |
| Extreme Type S | 164 | 4.00 | 33 | 1.00 |

*This table reports frequency of cognitive types in the YourMorals dataset.*

**Table S7. Means, SDs, and effect sizes across five moral foundations by cognitive type in dataset 2.**

|  | | Care | | | | Fairness | | |  | Loyalty | | | | Authority | | | | Sanctity | | | |
| --- | --- | --- | --- | --- | --- | --- | --- | --- | --- | --- | --- | --- | --- | --- | --- | --- | --- | --- | --- | --- | --- |
| Males | *N* | Mean | *SD* | *p* | *F* | Mean | *SD* | *p* | *F* | Mean | *SD* | *p* | *F* | Mean | *SD* | *p* | *F* | Mean | *SD* | *p* | *F* |
| Extreme Type E | 24 | 3.76 | 0.72 | < 3.7x10^-38^ | 46.34 | 3.28 | 0.85 | < 3.9x10^-6^ | 7.64 | 2.17 | 0.79 | .022 | 2.87 | 2.28 | 0.86 | .004 | 3.90 | 1.39 | 0.98 | 1.955 | .10 |
| Type E | 772 | 3.56 | 0.76 |  |  | 3.76 | 0.56 |  |  | 2.20 | 0.81 |  |  | 2.10 | 0.83 |  |  | 1.38 | 0.95 |  |  |
| Type B | 1295 | 3.36 | 0.83 |  |  | 3.68 | 0.69 |  |  | 2.25 | 0.84 |  |  | 2.22 | 0.87 |  |  | 1.43 | 1.04 |  |  |
| Type S | 1882 | 3.13 | 0.86 |  |  | 3.57 | 0.74 |  |  | 2.26 | 0.85 |  |  | 2.21 | 0.88 |  |  | 1.37 | 1.03 |  |  |
| Extreme Type S | 164 | 3.00 | 0.86 |  |  | 3.51 | 0.76 |  |  | 2.05 | 0.94 |  |  | 2.05 | 0.99 |  |  | 1.21 | 1.09 |  |  |
|  | | Care | | | | Fairness | | |  | Ingroup | | | | Authority | | | | Purity | | | |
| Females | *N* | Mean | *SD* | *p* | *F* | Mean | *SD* | *p* | *F* | Mean | *SD* | *p* | *F* | Mean | *SD* | *p* | *F* | Mean | *SD* | *p* | ***F*** |
| Extreme Type E | 159 | 4.09 | 0.55 | < 8.9x10^-42^ | 51.00 | 3.87 | 0.56 | < 6.1x10^-7^ | 8.65 | 2.03 | 0.72 | .062 | 2.24 | 1.98 | 0.82 | .073 | 2.14 | 1.32 | 0.85 | .000 | 5.35 |
| Type E | 1695 | 3.88 | 0.63 |  |  | 3.77 | 0.63 |  |  | 2.15 | 0.77 |  |  | 2.11 | 0.82 |  |  | 1.54 | 1.01 |  |  |
| Type B | 983 | 3.69 | 0.69 |  |  | 3.71 | 0.64 |  |  | 2.16 | 0.80 |  |  | 2.13 | 0.84 |  |  | 1.43 | 0.98 |  |  |
| Type S | 588 | 3.53 | 0.80 |  |  | 3.65 | 0.71 |  |  | 2.09 | 0.80 |  |  | 2.04 | 0.81 |  |  | 1.35 | 0.97 |  |  |
| Extreme Type S | 33 | 3.09 | 0.97 |  |  | 3.37 | 0.98 |  |  | 1.90 | 1.01 |  |  | 2.05 | 1.13 |  |  | 1.42 | 1.04 |  |  |

*This table provides the means, SDs, F statistics and the associated p values (MANOVAs) for the five moral foundations separately for males and females in dataset 2.*

**Table S8. Post hoc Tukey tests from MANOVAs for moral foundations by cognitive type in females in dataset 2.**

| Dependent Variable |  |  | Mean Difference (I-J) | Std. Error | Sig. | 95% Confidence Interval (lower bound) | 95% Confidence Interval (upper bound) |
| --- | --- | --- | --- | --- | --- | --- | --- |
| Care | Extreme Type E | Type E | 0.27^*^ | 0.06 | 0 | 0.05 | 0.36 |
|  |  | Type B | 0.40^*^ | 0.06 | 0 | 0.24 | 0.56 |
|  |  | Type S | 0.56^*^ | 0.06 | 0 | 0.4 | 0.73 |
|  |  | Extreme Type S | 0.99^*^ | 0.13 | 0 | 0.65 | 1.35 |
|  | Type E | Extreme Type E | -0.21^*^ | 0.06 | 0 | -0.36 | -0.05 |
|  |  | Type B | 0.19^*^ | 0.03 | 0 | 0.12 | 0.27 |
|  |  | Type S | 0.35^*^ | 0.03 | 0 | 0.27 | 0.44 |
|  |  | Extreme Type S | 0.79^*^ | 0.12 | 0 | 0.47 | 1.12 |
|  | Type B | Extreme Type E | -0.40^*^ | 0.06 | 0 | -0.56 | -0.24 |
|  |  | Type E | -0.19^*^ | 0.03 | 0 | -0.27 | -0.12 |
|  |  | Type S | 0.16^*^ | 0.04 | 0 | 0.06 | 0.26 |
|  |  | Extreme Type S | 0.60^*^ | 0.12 | 0 | 0.27 | 0.93 |
|  | Type S | Extreme Type E | -0.56^*^ | 0.06 | 0 | -0.73 | -0.4 |
|  |  | Type E | -0.35^*^ | 0.03 | 0 | -0.44 | -0.27 |
|  |  | Type B | -0.16^*^ | 0.04 | 0 | -0.26 | -0.06 |
|  |  | Extreme Type S | 0.44^*^ | 0.12 | 0 | 0.11 | 0.77 |
|  | Extreme Type S | Extreme Type E | -0.99^*^ | 0.13 | 0 | -1.35 | -0.65 |
|  |  | Type E | -0.79^*^ | 0.12 | 0 | -1.12 | -0.47 |
|  |  | Type B | -0.60^*^ | 0.12 | 0 | -0.93 | -0.27 |
|  |  | Type S | -0.44^*^ | 0.12 | 0 | -0.77 | -0.11 |
| Fairness | Extreme Type E | Type E | 0.1 | 0.05 | 0.34 | -0.05 | 0.25 |
|  |  | Type B | 0.16^*^ | 0.06 | 0.03 | 0.01 | 0.31 |
|  |  | Type S | 0.22^*^ | 0.06 | 0 | 0.07 | 0.38 |
|  |  | Extreme Type S | 0.49^*^ | 0.12 | 0 | 0.16 | 0.83 |
|  | Type E | Extreme Type E | -0.1 | 0.05 | 0.34 | -0.25 | 0.05 |
|  |  | Type B | 0.06 | 0.03 | 0.15 | -0.01 | 0.13 |
|  |  | Type S | 0.12^*^ | 0.03 | 0 | 0.04 | 0.21 |
|  |  | Extreme Type S | 0.39^*^ | 0.11 | 0.01 | 0.09 | 0.7 |
|  | Type B | Extreme Type E | -.016^*^ | 0.06 | 0.03 | -0.31 | -0.01 |
|  |  | Type E | -0.06 | 0.03 | 0.15 | -0.13 | 0.01 |
|  |  | Type S | 0.06 | 0.03 | 0.31 | -0.03 | 0.16 |
|  |  | Extreme Type S | 0.33^*^ | 0.11 | 0.03 | 0.02 | 0.65 |
|  | Type S | Extreme Type E | -0.22^*^ | 0.06 | 0 | -0.38 | -0.07 |
|  |  | Type E | -0.12^*^ | 0.03 | 0 | -0.21 | -0.04 |
|  |  | Type B | -0.06 | 0.03 | 0.31 | -0.16 | 0.03 |
|  |  | Extreme Type S | 0.27 | 0.12 | 0.13 | -0.04 | 0.59 |
|  | Extreme Type S | Extreme Type E | -0.49^*^ | 0.12 | 0 | -0.83 | -0.16 |
|  |  | Type E | -.039^*^ | 0.11 | 0.01 | -0.7 | -0.09 |
|  |  | Type B | -0.34^*^ | 0.11 | 0.03 | -0.65 | -0.02 |
|  |  | Type S | -0.27 | 0.12 | 0.13 | -0.59 | 0.04 |
| Loyalty | Extreme Type E | Type E | -0.12 | 0.07 | 0.32 | -0.3 | 0.05 |
|  |  | Type B | -0.12 | 0.07 | 0.34 | -0.31 | 0.06 |
|  |  | Type S | -0.06 | 0.07 | 0.89 | -0.26 | 0.13 |
|  |  | Extreme Type S | 0.13 | 0.15 | 0.91 | -0.28 | 0.54 |
|  | Type E | Extreme Type E | 0.12 | 0.07 | 0.32 | -0.05 | 0.3 |
|  |  | Type B | 0 | 0.03 | 1 | -0.09 | 0.08 |
|  |  | Type S | 0.06 | 0.04 | 0.51 | -0.04 | 0.16 |
|  |  | Extreme Type S | 0.25 | 0.14 | 0.35 | -0.12 | 0.63 |
|  | Type B | Extreme Type E | 0.12 | 0.07 | 0.34 | -0.06 | 0.31 |
|  |  | Type E | 0 | 0.03 | 1 | -0.08 | 0.09 |
|  |  | Type S | 0.06 | 0.04 | 0.57 | -0.05 | 0.17 |
|  |  | Extreme Type S | 0.26 | 0.14 | 0.35 | -0.12 | 0.64 |
|  | Type S | Extreme Type E | 0.06 | 0.07 | 0.89 | -0.13 | 0.26 |
|  |  | Type E | -0.06 | 0.04 | 0.51 | -0.16 | 0.04 |
|  |  | Type B | -0.06 | 0.04 | 0.57 | -0.17 | 0.05 |
|  |  | Extreme Type S | 0.2 | 0.14 | 0.64 | -0.19 | 0.58 |
|  | Extreme Type S | Extreme Type E | -0.13 | 0.15 | 0.91 | -0.54 | 0.28 |
|  |  | Type E | -0.25 | 0.14 | 0.35 | -0.63 | 0.12 |
|  |  | Type B | -0.26 | 0.14 | 0.35 | -0.64 | 0.12 |
|  |  | Type S | -0.2 | 0.14 | 0.64 | -0.58 | 0.19 |
| Authority | Extreme Type E | Type E | -0.13 | 0.07 | 0.3 | -0.32 | 0.05 |
|  |  | Type B | -0.16 | 0.07 | 0.17 | -0.35 | 0.04 |
|  |  | Type S | -0.06 | 0.07 | 0.91 | -0.26 | 0.14 |
|  |  | Extreme Type S | -0.08 | 0.16 | 0.99 | -0.51 | 0.35 |
|  | Type E | Extreme Type E | 0.13 | 0.07 | 0.3 | -0.05 | 0.32 |
|  |  | Type B | -0.02 | 0.03 | 0.95 | -0.11 | 0.07 |
|  |  | Type S | 0.07 | 0.04 | 0.4 | -0.04 | 0.18 |
|  |  | Extreme Type S | 0.06 | 0.14 | 1 | -0.34 | 0.45 |
|  | Type B | Extreme Type E | 0.16 | 0.07 | 0.17 | -0.04 | 0.35 |
|  |  | Type E | 0.02 | 0.03 | 0.95 | -0.07 | 0.11 |
|  |  | Type S | 0.09 | 0.04 | 0.19 | -0.02 | 0.21 |
|  |  | Extreme Type S | 0.08 | 0.15 | 0.98 | -0.32 | 0.48 |
|  | Type S | Extreme Type E | 0.06 | 0.07 | 0.91 | -0.14 | 0.26 |
|  |  | Type E | -0.07 | 0.04 | 0.4 | -0.18 | 0.04 |
|  |  | Type B | -0.09 | 0.04 | 0.19 | -0.21 | 0.02 |
|  |  | Extreme Type S | -0.01 | 0.15 | 1 | -0.41 | 0.39 |
|  | Extreme Type S | Extreme Type E | 0.08 | 0.16 | 0.99 | -0.35 | 0.51 |
|  |  | Type E | -0.06 | 0.14 | 1 | -0.45 | 0.34 |
|  |  | Type B | -0.08 | 0.15 | 0.98 | -0.48 | 0.32 |
|  |  | Type S | 0.01 | 0.15 | 1 | -0.39 | 0.41 |
| Sanctity | Extreme Type E | Type E | -0.22 | 0.08 | 0.07 | -0.44 | 0.01 |
|  |  | Type B | -0.11 | 0.08 | 0.68 | -0.34 | 0.12 |
|  |  | Type S | -0.03 | 0.09 | 1 | -0.27 | 0.21 |
|  |  | Extreme Type S | -0.1 | 0.19 | 0.99 | -0.61 | 0.42 |
|  | Type E | Extreme Type E | 0.22 | 0.08 | 0.07 | -0.01 | 0.44 |
|  |  | Type B | 0.1 | 0.04 | 0.06 | 0 | 0.21 |
|  |  | Type S | 0.19^*^ | 0.05 | 0 | 0.06 | 0.32 |
|  |  | Extreme Type S | 0.12 | 0.17 | 0.96 | -0.36 | 0.59 |
|  | Type B | Extreme Type E | 0.11 | 0.08 | 0.68 | -0.12 | 0.34 |
|  |  | Type E | -0.1 | 0.04 | 0.06 | -0.21 | 0 |
|  |  | Type S | 0.08 | 0.05 | 0.51 | -0.06 | 0.22 |
|  |  | Extreme Type S | 0.01 | 0.17 | 1 | -0.46 | 0.49 |
|  | Type S | Extreme Type E | 0.03 | 0.09 | 1 | -0.21 | 0.27 |
|  |  | Type E | -.019^*^ | 0.05 | 0 | -0.32 | -0.06 |
|  |  | Type B | -0.08 | 0.05 | 0.51 | -0.22 | 0.06 |
|  |  | Extreme Type S | -0.07 | 0.18 | 1 | -0.55 | 0.41 |
|  | Extreme Type S | Extreme Type E | 0.1 | 0.19 | 0.99 | -0.42 | 0.61 |
|  |  | Type E | -0.12 | 0.17 | 0.96 | -0.59 | 0.36 |
|  |  | Type B | -0.01 | 0.17 | 1 | -0.49 | 0.46 |
|  |  | Type S | 0.07 | 0.18 | 1 | -0.41 | 0.55 |
|  |  |  |  |  |  |  | 1 |

*This table reports results post-hoc Tukey tests.*

**Table S9. Post hoc Tukey tests from MANOVAs for moral foundations by cognitive type in males in Study 2**

| Dependent Variable |  |  | Mean Difference (I-J) | Std. Error | Sig. | 95% Confidence Interval (lower bound) | 95% Confidence Interval (upper bound) |  |
| --- | --- | --- | --- | --- | --- | --- | --- | --- |
| Care | Extreme Type E | Type E | 0.2 | 0.17 | 0.76 | -0.27 | 0.68 |  |
|  |  | Type B | 0.41 | 0.17 | 0.12 | -0.06 | 0.88 |  |
|  |  | Type S | 0.63^*^ | 0.17 | 0 | 0.17 | 1.1 |  |
|  |  | Extreme Type S | 0.76^*^ | 0.18 | 0 | 0.26 | 1.26 |  |
|  | Type E | Extreme Type E | -0.2 | 0.17 | 0.76 | -0.68 | 0.27 |  |
|  |  | Type B | 0.20^*^ | 0.04 | 0 | 0.1 | 0.31 |  |
|  |  | Type S | 0.43^*^ | 0.04 | 0 | 0.33 | 0.53 |  |
|  |  | Extreme Type S | 0.56^*^ | 0.07 | 0 | 0.36 | 0.75 |  |
|  | Type B | Extreme Type E | -0.41 | 0.17 | 0.12 | -0.88 | 0.06 |  |
|  |  | Type E | -0.20^*^ | 0.04 | 0 | -0.31 | -0.1 |  |
|  |  | Type S | 0.23^*^ | 0.03 | 0 | 0.15 | 0.31 |  |
|  |  | Extreme Type S | 0.35^*^ | 0.07 | 0 | 0.16 | 0.54 |  |
|  | Type S | Extreme Type E | -0.64^*^ | 0.17 | 0 | -1.1 | -0.17 |  |
|  |  | Type E | -0.43^*^ | 0.04 | 0 | -0.53 | -0.33 |  |
|  |  | Type B | -0.24^*^ | 0.03 | 0 | -0.31 | -0.15 |  |
|  |  | Extreme Type S | 0.12 | 0.07 | 0.36 | -0.06 | 0.31 |  |
|  | Extreme Type S | Extreme Type E | -0.76^*^ | 0.18 | 0 | -1.26 | -0.26 |  |
|  |  | Type E | -0.56^*^ | 0.07 | 0 | -0.75 | -0.36 |  |
|  |  | Type B | -0.35^*^ | 0.07 | 0 | -0.54 | -0.16 |  |
|  |  | Type S | -0.12 | 0.07 | 0.36 | -0.31 | 0.06 |  |
| Fairness | Extreme Type E | Type E | 0.09 | 0.15 | 0.98 | -0.33 | 0.51 |  |
|  |  | Type B | 0.19 | 0.15 | 0.71 | -0.22 | 0.61 |  |
|  |  | Type S | 0.26 | 0.15 | 0.45 | -0.16 | 0.67 |  |
|  |  | Extreme Type S | 0.2 | 0.16 | 0.72 | -0.24 | 0.65 |  |
|  | Type E | Extreme Type E | -0.09 | 0.15 | 0.98 | -0.51 | 0.33 |  |
|  |  | Type B | 0.11^*^ | 0.03 | 0.01 | 0.02 | 0.2 |  |
|  |  | Type S | 0.17^*^ | 0.03 | 0 | 0.08 | 0.26 |  |
|  |  | Extreme Type S | 0.12 | 0.06 | 0.35 | -0.06 | 0.29 |  |
|  | Type B | Extreme Type E | -0.19 | 0.15 | 0.71 | -0.61 | 0.22 |  |
|  |  | Type E | -0.11^*^ | 0.03 | 0.01 | -0.2 | -0.02 |  |
|  |  | Type S | 0.06 | 0.03 | 0.14 | -0.01 | 0.14 |  |
|  |  | Extreme Type S | 0.01 | 0.06 | 1 | -0.16 | 0.18 |  |
|  | Type S | Extreme Type E | -0.26 | 0.15 | 0.45 | -0.67 | 0.16 |  |
|  |  | Type E | -0.17^*^ | 0.03 | 0 | -0.26 | -0.08 |  |
|  |  | Type B | -0.06 | 0.03 | 0.14 | -0.14 | 0.01 |  |
|  |  | Extreme Type S | -0.05 | 0.06 | 0.91 | -0.22 | 0.11 |  |
|  | Extreme Type S | Extreme Type E | -0.2 | 0.16 | 0.72 | -0.65 | 0.24 |  |
|  |  | Type E | -0.12 | 0.06 | 0.35 | -0.29 | 0.06 |  |
|  |  | Type B | -0.01 | 0.06 | 1 | -0.18 | 0.16 |  |
|  |  | Type S | 0.05 | 0.06 | 0.91 | -0.11 | 0.22 |  |
| Loyalty | Extreme Type E | Type E | -0.04 | 0.17 | 1 | -0.51 | 0.44 |  |
|  |  | Type B | -0.08 | 0.17 | 0.99 | -0.56 | 0.39 |  |
|  |  | Type S | -0.09 | 0.17 | 0.98 | -0.56 | 0.38 |  |
|  |  | Extreme Type S | 0.12 | 0.18 | 0.96 | -0.38 | 0.62 |  |
|  | Type E | Extreme Type E | 0.04 | 0.17 | 1 | -0.44 | 0.51 |  |
|  |  | Type B | -0.04 | 0.04 | 0.77 | -0.15 | 0.06 |  |
|  |  | Type S | -0.05 | 0.04 | 0.55 | -0.15 | 0.04 |  |
|  |  | Extreme Type S | 0.16 | 0.07 | 0.18 | -0.04 | 0.36 |  |
|  | Type B | Extreme Type E | 0.08 | 0.17 | 0.99 | -0.39 | 0.56 |  |
|  |  | Type E | 0.04 | 0.04 | 0.77 | -0.06 | 0.15 |  |
|  |  | Type S | -0.01 | 0.03 | 1 | -0.09 | 0.07 |  |
|  |  | Extreme Type S | 00.20^*^ | 0.07 | 0.03 | 0.01 | 0.39 |  |
|  | Type S | Extreme Type E | 0.09 | 0.17 | 0.98 | -0.38 | 0.56 |  |
|  |  | Type E | 0.05 | 0.04 | 0.55 | -0.04 | 0.15 |  |
|  |  | Type B | 0.01 | 0.03 | 1 | -0.07 | 0.09 |  |
|  |  | Extreme Type S | 0.21^*^ | 0.07 | 0.02 | 0.03 | 0.4 |  |
|  | Extreme Type S | Extreme Type E | -0.12 | 0.18 | 0.96 | -0.62 | 0.38 |  |
|  |  | Type E | -0.16 | 0.07 | 0.18 | -0.36 | 0.04 |  |
|  |  | Type B | -0.20^*^ | 0.07 | 0.03 | -0.39 | -0.01 |  |
|  |  | Type S | -0.21^*^ | 0.07 | 0.02 | -0.4 | -0.03 |  |
| Authority | Extreme Type E | Type E | 0.18 | 0.18 | 0.86 | -0.32 | 0.67 |  |
|  |  | Type B | 0.05 | 0.18 | 1 | -0.44 | 0.54 |  |
|  |  | Type S | 0.07 | 0.18 | 1 | -0.42 | 0.56 |  |
|  |  | Extreme Type S | 0.23 | 0.19 | 0.75 | -0.29 | 0.75 |  |
|  | Type E | Extreme Type E | -0.18 | 0.18 | 0.86 | -0.67 | 0.32 |  |
|  |  | Type B | -0.12^*^ | 0.04 | 0.02 | -0.23 | -0.02 |  |
|  |  | Type S | -0.11^*^ | 0.04 | 0.03 | -0.21 | -0.01 |  |
|  |  | Extreme Type S | 0.05 | 0.07 | 0.96 | -0.15 | 0.26 |  |
|  | Type B | Extreme Type E | -0.05 | 0.18 | 1 | -0.54 | 0.44 |  |
|  |  | Type E | 0.12^*^ | 0.04 | 0.02 | 0.02 | 0.23 |  |
|  |  | Type S | 0.02 | 0.03 | 0.99 | -0.07 | 0.1 |  |
|  |  | Extreme Type S | 0.18 | 0.07 | 0.11 | -0.02 | 0.37 |  |
|  | Type S | Extreme Type E | -0.07 | 0.18 | 1 | -0.56 | 0.42 |  |
|  |  | Type E | 0.11^*^ | 0.04 | 0.03 | 0.01 | 0.21 |  |
|  |  | Type B | -0.02 | 0.03 | 0.99 | -0.1 | 0.07 |  |
|  |  | Extreme Type S | 0.16 | 0.07 | 0.16 | -0.03 | 0.35 |  |
|  | Extreme Type S | Extreme Type E | -0.23 | 0.19 | 0.75 | -0.75 | 0.29 |  |
|  |  | Type E | -0.05 | 0.07 | 0.96 | -0.26 | 0.15 |  |
|  |  | Type B | -0.18 | 0.07 | 0.11 | -0.37 | 0.02 |  |
|  |  | Type S | -0.16 | 0.07 | 0.16 | -0.35 | 0.03 |  |
| Sanctity | Extreme Type E | Type E | 0.01 | 0.21 | 1 | -0.57 | 0.59 |  |
|  |  | Type B | -0.04 | 0.21 | 1 | -0.62 | 0.53 |  |
|  |  | Type S | 0.01 | 0.21 | 1 | -0.56 | 0.59 |  |
|  |  | Extreme Type S | 0.18 | 0.22 | 0.93 | -0.43 | 0.79 |  |
|  | Type E | Extreme Type E | -0.01 | 0.21 | 1 | -0.59 | 0.57 |  |
|  |  | Type B | -0.06 | 0.05 | 0.75 | -0.18 | 0.07 |  |
|  |  | Type S | 0 | 0.04 | 1 | -0.12 | 0.12 |  |
|  |  | Extreme Type S | 0.16 | 0.09 | 0.33 | -0.08 | 0.4 |  |
|  | Type B | Extreme Type E | 0.04 | 0.21 | 1 | -0.53 | 0.62 |  |
|  |  | Type E | 0.06 | 0.05 | 0.75 | -0.07 | 0.18 |  |
|  |  | Type S | 0.06 | 0.04 | 0.5 | -0.04 | 0.16 |  |
|  |  | Extreme Type S | 0.22 | 0.08 | 0.07 | -0.01 | 0.45 |  |
|  | Type S | Extreme Type E | -0.01 | 0.21 | 1 | -0.59 | 0.56 |  |
|  |  | Type E | 0 | 0.04 | 1 | -0.12 | 0.12 |  |
|  |  | Type B | -0.06 | 0.04 | 0.5 | -0.16 | 0.04 |  |
|  |  | Extreme Type S | 0.16 | 0.08 | 0.29 | -0.07 | 0.39 |  |
|  | Extreme Type S | Extreme Type E | -0.18 | 0.22 | 0.93 | -0.79 | 0.43 |  |
|  |  | Type E | -0.16 | 0.09 | 0.33 | -0.4 | 0.08 |  |
|  |  | Type B | -0.22 | 0.08 | 0.07 | -0.45 | 0.01 |  |
|  |  | Type S | -0.16 | 0.08 | 0.29 | -0.39 | 0.07 |  |
|  |  |  |  |  |  |  |  |  |

*This table reports results post hoc Tukey tests.*

**Table S10. Results from linear regressions, with cognitive empathy, affective empathy, and SQ scores predicting Care and Fairness, separately for females and males in dataset 2.**

| Care | | | | | | | | | | |
| --- | --- | --- | --- | --- | --- | --- | --- | --- | --- | --- |
|  | females | | | | | males | | | | |
|  | unstandardized B | Std. Error | Standardized Beta | *t* | Sig. | unstandardized B | Std. Error | Standardized Beta | *t* | Sig. |
| (Constant) | 1.94 | 0.20 |  | 9.83 | < .001 | 1.43 | 0.21 |  | 6.70 | < .001 |
| Cognitive Empathy | -0.01 | 0.05 | -0.01 | -0.30 | 0.77 | -0.01 | 0.05 | -0.01 | -0.16 | 0.88 |
| Affective Empathy | 0.49 | 0.04 | 0.52 | 11.28 | < .001 | 0.62 | 0.05 | 0.56 | 13.22 | < .001 |
| SQ | -0.01 | 0.01 | -0.04 | -1.00 | 0.32 | -0.02 | 0.01 | -0.14 | -3.55 | < .001 |
|  | *r* =.52; *r*^2^ = .27 | | | | | *r* =.57; *r*^2^ = .31 | | | | |
| Fairness | | | | | | | | | | |
|  | females | | | | |  |  |  |  |  |
|  | unstandardized B | Std. Error | Standardized Beta | *t* | Sig. | unstandardized B | Std. Error | Standardized Beta | *t* | Sig. |
| (Constant) | 2.66 | 0.20 |  | 13.23 | < .001 | 2.23 | 0.21 |  | 10.82 | < .001 |
| Cognitive Empathy | 0.00 | 0.05 | 0.00 | -0.06 | 0.95 | -0.05 | 0.05 | -0.05 | -1.03 | 0.30 |
| Affective Empathy | 0.27 | 0.04 | 0.31 | 6.06 | < .001 | 0.44 | 0.05 | 0.45 | 9.60 | < .001 |
| SQ | 0.00 | 0.01 | 0.02 | 0.32 | 0.75 | 0.00 | 0.01 | -0.03 | -0.65 | 0.52 |
|  | *r* = .31; *r*^2^ = .09 | | | | | *r* = .43; *r*^2^ = .19 | | | | |

*N*s = 424 for females and 471 for males.

**Table S11. Mean D-scores for each political category in dataset 2.**

| Females | | | | | | |
| --- | --- | --- | --- | --- | --- | --- |
|  | *N* | *M* | *SD* | *STE* | 95% Cis | |
| Liberals | 2726 | -7.5 | 13.3 | 0.3 | -8.1 | -7.0 |
| Moderates | 282 | -5.6 | 13.6 | 0.8 | -7.2 | -4.1 |
| Conservatives | 205 | -5.4 | 14.4 | 1.0 | -7.4 | -3.5 |
| Libertarians | 111 | 2.6 | 13.2 | 1.3 | 0.1 | 5.1 |
| Total | 3324 | -6.9 | 13.6 | 0.2 | -7.4 | -6.5 |
| Males | | | | | | |
|  | *N* | *M* | *SD* | *STE* | 95% CIs | |
| Liberals | 2670 | 4.00 | 13.85 | 0.27 | 3.47 | 4.53 |
| Moderates | 348 | 6.34 | 12.88 | 0.69 | 4.98 | 7.70 |
| Conservatives | 434 | 6.68 | 12.82 | 0.62 | 5.47 | 7.89 |
| Libertarians | 414 | 11.18 | 13.09 | 0.64 | 9.92 | 12.45 |
| Total | 3866 | 5.28 | 13.76 | 0.22 | 4.85 | 5.71 |

**Table S12. D-scores and cognitive types in dataset 2.**

| Females | | | | | | |
| --- | --- | --- | --- | --- | --- | --- |
|  |  | Mean Difference (I-J) | STE | *p* | 95% CI | |
| Liberals | Moderates | -1.90616 | 0.84 | 0.11 | -4.07 | 0.25 |
|  | Conservatives | -2.11112 | 0.97 | 0.13 | -4.61 | 0.39 |
|  | Libertarians | -10.12369* | 1.30 | 0.00 | -13.47 | -6.78 |
| Moderates | Liberals | 1.90616 | 0.84 | 0.11 | -0.25 | 4.07 |
|  | Conservatives | -0.20497 | 1.23 | 1.00 | -3.37 | 2.96 |
|  | Libertarians | -8.21753* | 1.50 | 0.00 | -12.09 | -4.35 |
| Conservatives | Liberals | 2.11112 | 0.97 | 0.13 | -0.39 | 4.61 |
|  | Moderates | 0.20497 | 1.23 | 1.00 | -2.96 | 3.37 |
|  | Libertarians | -8.01257* | 1.58 | 0.00 | -12.08 | -3.94 |
| Libertarians | Liberals | 10.12369* | 1.30 | 0.00 | 6.78 | 13.47 |
|  | Moderates | 8.21753* | 1.50 | 0.00 | 4.35 | 12.09 |
|  | Conservatives | 8.01257* | 1.58 | 0.00 | 3.94 | 12.08 |
| Males | | | | | | |
|  |  | Mean Difference (I-J) | STE | *p* | 95% CI | |
| Liberals | Moderates | -2.33815* | 0.77 | 0.01 | -4.33 | -0.35 |
|  | Conservatives | -2.67964* | 0.70 | 0.00 | -4.49 | -0.87 |
|  | Libertarians | -7.18191* | 0.72 | 0.00 | -9.02 | -5.34 |
| Moderates | Liberals | 2.33815* | 0.77 | 0.01 | 0.35 | 4.33 |
|  | Conservatives | -0.3415 | 0.98 | 0.99 | -2.85 | 2.17 |
|  | Libertarians | -4.84376* | 0.99 | 0.00 | -7.38 | -2.31 |
| Conservatives | Liberals | 2.67964* | 0.70 | 0.00 | 0.87 | 4.49 |
|  | Moderates | 0.3415 | 0.98 | 0.99 | -2.17 | 2.85 |
|  | Libertarians | -4.50226* | 0.93 | 0.00 | -6.90 | -2.11 |
| Libertarians | Liberals | 7.18191* | 0.72 | 0.00 | 5.34 | 9.02 |
|  | Moderates | 4.84376* | 0.99 | 0.00 | 2.31 | 7.38 |
|  | Conservatives | 4.50226* | 0.93 | 0.00 | 2.11 | 6.90 |

**Table S13. Frequency of political identification within each cognitive type in dataset 2.**

| Cognitive type | | Liberal | | | Moderate | | | Conservative | | | Libertarian | | |
| --- | --- | --- | --- | --- | --- | --- | --- | --- | --- | --- | --- | --- | --- |
| Label | *n (cognitive type)* | *n (liberal)* | % PI | % BT | *n (moderate)* | % PI | % BT | *n (conservative)* | % PI | % BT | *n (libertarian)* | % PI | % BT |
| Extreme Type E | 154 | 138 | 5% | 90% | 8 | 3% | 6% | 7 | 3% | 5% | 1 | 0% | 1% |
| Type E | 1647 | 1385 | 51% | 84% | 137 | 49% | 10% | 100 | 49% | 6% | 25 | 12% | 2% |
| Type B | 942 | 758 | 28% | 80% | 81 | 29% | 11% | 57 | 28% | 6% | 46 | 22% | 5% |
| Type S | 550 | 424 | 16% | 77% | 53 | 19% | 13% | 37 | 18% | 7% | 36 | 18% | 7% |
| Extreme Type S | 31 | 21 | 1% | 68% | 3 | 1% | 14% | 4 | 2% | 13% | 3 | 1% | 10% |
| Total: | 3324 | 2726 | 100% |  | 282 | 100% |  | 205 | 100% |  | 205 | 100% |  |
| Cognitive type | | Liberal | | | Moderate | | | Conservative | | | Libertarian | | |
| Label | *n (cognitive type)* | *n (liberal)* | % PI | % BT | *n (moderate)* | % PI | % BT | *n (conservative)* | % PI | % BT | *n (libertarian)* | % PI | % BT |
|  | *N* | Liberal | % PI | % BT | Moderate | % PI | % BT | Conservative | % PI | % BT | Libertarian | % PI | % BT |
| Extreme Type E | 24 | 19 | 1% | 79% | 4 | 1% | 17% | 1 | 0% | 4% | 0 | 0% | 0% |
| Type E | 734 | 586 | 22% | 80% | 51 | 15% | 7% | 62 | 14% | 8% | 35 | 8% | 5% |
| Type B | 1210 | 869 | 33% | 72% | 105 | 30% | 9% | 143 | 33% | 12% | 93 | 22% | 8% |
| Type S | 1749 | 1101 | 41% | 63% | 174 | 50% | 10% | 213 | 49% | 12% | 261 | 63% | 15% |
| Extreme Type S | 149 | 95 | 4% | 64% | 14 | 4% | 9% | 15 | 3% | 10% | 25 | 6% | 17% |
| Total: | 3866 | 2670 | 100% |  | 348 | 100% |  | 434 |  |  | 414 | 100% |  |
